# Pharmacologic Modulation of ARID3A with Rimegepant Reactivates Type I Interferon Signaling and Sensitizes Triple‐Negative Breast Cancer to PD‐1 Blockade

**DOI:** 10.1002/advs.202521541

**Published:** 2026-06-02

**Authors:** Teng Zhou, Yifei Zhu, Cheng Zeng, Jinlu Han, Huiying Huang, Doudou Li, Mingxi Lin, Yizi Jin, Qin Guo, Yuxin Yan, Xinhui Mao, Yifei Zhou, Jian Zhang

**Affiliations:** ^1^ Phase I Unit Fudan University Shanghai Cancer Center Shanghai China; ^2^ Department of Oncology Shanghai Medical College Fudan University Shanghai China; ^3^ Department of Pancreatic Surgery Fudan University Shanghai Cancer Center Shanghai China; ^4^ Department of Gastroenterology Tongren Hospital Shanghai Jiao Tong University School of Medicine Shanghai China; ^5^ ENT Institute and Department of Otorhinolaryngology Eye & ENT Hospital Fudan University Shanghai China; ^6^ School of Clinical Medicine Shanghai University of Medicine & Health Sciences Shanghai China; ^7^ Department of Thoracic Surgery Shanghai Pulmonary Hospital Tongji University School of Medicine Shanghai China

**Keywords:** ARID3A, immune checkpoint blockade, rimegepant, triple‐negative breast cancer, Type I interferon pathway

## Abstract

Immune checkpoint blockade (ICB) shows limited clinical activity in TNBC, largely because immune‐cold tumors fail to sustain productive innate and adaptive anti‐tumor immunity within an immunosuppressive tumor microenvironment. Here, we identify the transcription factor ARID3A as a tumor‐intrinsic regulator of this resistant state. Genetic ablation of ***ARID3A*** inflamed the tumor microenvironment, enhanced dendritic‐cell activation, increased antigen‐specific tetramer‐positive and total CD8^+^ T‐cell infiltration with heightened cytotoxic activity, and sensitized otherwise resistant tumors to PD‐1 blockade in vivo. Mechanistically, ARID3A restrained endogenous nucleic‐acid sensing and downstream type I interferon signaling, predominantly through cGAS‐STING with a contributory RIG‐I‐MAVS component. Through structure‐guided drug repurposing, we identified the clinically approved migraine agent rimegepant as an ARID3A‐targeting small molecule. Direct binding assays, together with binding‐pocket mutations (Y326A and R266A) in matched‐expression rescue systems, provided strong genetic evidence for on‐target engagement of rimegepant with ARID3A. Rimegepant phenocopied ***ARID3A*** loss, reactivated innate immune signaling, and markedly enhanced anti‐PD‐1 efficacy in murine TNBC models and patient‐derived organoids. Collectively, our findings define an actionable ARID3A‐IFN axis and support clinical evaluation of rimegepant‐based immunotherapy combinations, particularly in ARID3A‐high, immune‐cold TNBC.

## Graphical abstract

This study identifies ARID3A as a key immunosuppressive transcription factor in TNBC. Its inhibition activates the type I IFN pathway, boosting CD8^+^ T cell infiltration and sensitizing tumors to anti‐PD‐1. The FDA‐approved migraine drug Rimegepant targets ARID3A, enhances immunotherapy efficacy in preclinical models, and establishes a druggable axis to overcome resistance.

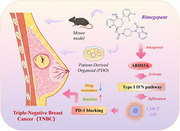

**Abbreviations**
breast cancer resistance protein: BCRP
Cancer Cell Line Encyclopedia: CCLE
Chimeric Antigen Receptor: CAR
distant metastasis‐free survival: DMFS
double‐stranded DNA: dsDNA
double‐stranded RNA: dsRNA
effector‐to‐target: E:T
estrogen receptor: ER
fluorescence‐activated cell sorting: FACS
Gene Ontology: GO
Gene Set Enrichment Analysis: GSEA
glioblastoma: GBM
granzyme B: GZMB
hepatocellular carcinoma: HCC
human epidermal growth factor receptor 2: HER2
Human Equivalent Dose: HED
immune checkpoint blockade: ICB
immunohistochemistry: IHC
Interferon‐stimulated gene: ISG
kidney renal clear cell carcinoma: KIRC
lung adenocarcinoma: LUAD
overall survival: OS
pathological complete responders: pCR
P‐glycoprotein: P‐gp
progesterone receptor: PR
progression‐free survival: PFS
skin cutaneous melanoma: SKCM
stomach adenocarcinoma: STAD
The Cancer Genome Atlas: TCGA
triple‐negative breast cancer: TNBC
Tumor Immune Dysfunction and Exclusion: TIDE
tumor microenvironment: TME
type I interferon: IFN‐I

## 1 Introduction

Triple‐negative breast cancer (TNBC)—defined by the absence of estrogen receptor (ER), progesterone receptor (PR), and human epidermal growth factor receptor 2 (HER2) amplification—is the most aggressive breast cancer subtype and accounts for ∼15% of all breast cancers [1]. Compared with other breast cancer subtypes, TNBC is associated with higher recurrence rates, earlier metastasis, and worse overall survival. The lack of actionable molecular targets has largely limited systemic therapy to cytotoxic chemotherapy; however, clinical benefits are often modest and transient, leaving a critical unmet need.

Immune checkpoint blockade (ICB) targeting the PD‐1/PD‐L1 axis has reshaped cancer therapy and is increasingly explored in breast cancer [2, 3]. Recent reviews highlight the rapid evolution of immunotherapy strategies in this field [4]. In TNBC, clinical trials have demonstrated activity of ICB in both early and advanced disease, leading to approvals in combination with chemotherapy [5, 6]. However, only a subset of patients derives durable benefit. Commonly used biomarkers, including PD‐L1 expression, show variable predictive value across settings and do not consistently identify responders [1, 7, 8]. A growing body of evidence suggests that resistance to ICB is closely linked to an immunosuppressive tumor microenvironment (TME), particularly immune‐cold states characterized by limited T‐cell infiltration. Such TMEs often correspond to immune “desert” or “excluded” phenotypes and are widely recognized as major barriers to effective immunotherapy [9]. These observations emphasize the importance of tumor‐intrinsic mechanisms that influence immune recognition and immune cell recruitment [10].

Among proposed strategies to overcome immune‐cold TMEs, activation of innate immune‐sensing pathways within tumor cells has attracted increasing attention. The cGAS–STING pathway, a central mediator of type I interferon responses, has been implicated in promoting dendritic cell activation and antitumor T‐cell priming. Modulation of this pathway is therefore considered a potential approach to enhance tumor immunogenicity and improve responses to ICB [11]. Nevertheless, the transcriptional regulators that constrain or permit activation of such innate immune programs in TNBC remain incompletely understood. Previous studies have linked immune‐cold TNBC to diverse mechanisms, including metabolic competition and T‐cell dysfunction, yet the upstream transcriptional factors coordinating these processes are not well defined [12]. Systematic identification of such regulators may provide insights into immune phenotype specification and reveal new therapeutic entry points.

AT‐rich interactive domain‐containing protein 3A (ARID3A), a member of the ARID family of transcription factors, regulates chromatin organization, differentiation, and cell fate decisions [13, 14, 15]. ARID family proteins have been implicated in immune cell development and interferon‐related signaling. Notably, ARID3A itself has been shown to regulate B‐cell lineage commitment and interferon responses [16]. The function of ARID3A in cancer appears to be context dependent. In several malignancies, including pancreatic [17] and liver cancers [18], ARID3A has been associated with tumor progression and therapeutic resistance through effects on ferroptosis, stemness, or transcriptional regulation [19]. Whether ARID3A exerts distinct roles in tumor immunogenicity or TME composition, particularly in TNBC, remains unclear. This uncertainty defines an important gap in current knowledge.

In parallel, there is interest in identifying clinically feasible strategies to modulate tumor‐intrinsic immune regulators. Drug repurposing offers a pragmatic approach by leveraging established safety and pharmacokinetic profiles. Using structure‐based virtual screening targeting the predicted DNA‐binding domain of ARID3A, we identified small molecules with the potential to interact with ARID3A. One such candidate is rimegepant, an orally available, FDA‐approved antagonist of the calcitonin gene‐related peptide (CGRP) receptor, currently used for the acute and preventive treatment of migraine [20]. Existing studies of rimegepant have primarily focused on its pharmacokinetics and drug–drug interactions, including its status as a substrate of P‐glycoprotein and breast cancer resistance protein [21, 22]. To date, rimegepant has not been linked to ARID3A regulation or tumor immune modulation.

In this study, we aimed to (i) examine whether ARID3A influences tumor cell immunogenicity and the immune landscape of TNBC, (ii) explore potential downstream mechanisms, including innate immune signaling pathways, and (iii) assess whether targeting this axis with rimegepant can reactivate innate immune signaling, including the cGAS–STING pathway, and synergize with immune checkpoint blockade to overcome therapeutic resistance.

## 2 Results

### 2.1 ARID3A is Upregulated in Breast Cancer and Correlates with an Immunosuppressive Phenotype

To identify tumor‐intrinsic regulators of early immune editing, we employed a four‐tiered screening strategy adapted from a prior study on ITPRIPL1 [23]. This approach integrates complementary immune‐related features to capture genes associated with immune tolerance, evasion, and impaired interferon responses. Specifically, we defined four gene sets: (i) genes positively correlated with immune‐privilege signatures (promoting local tolerance); (ii) genes negatively correlated with PD‐L1 (CD274) expression (identifying checkpoint‐independent evasion mechanisms); (iii) genes negatively associated with type I interferon (IFN‐I) signaling (critical for innate immunity); and (iv) genes negatively associated with type II interferon (IFN‐γ) signaling (essential for T cell–mediated immunity).

**FIGURE 1:**
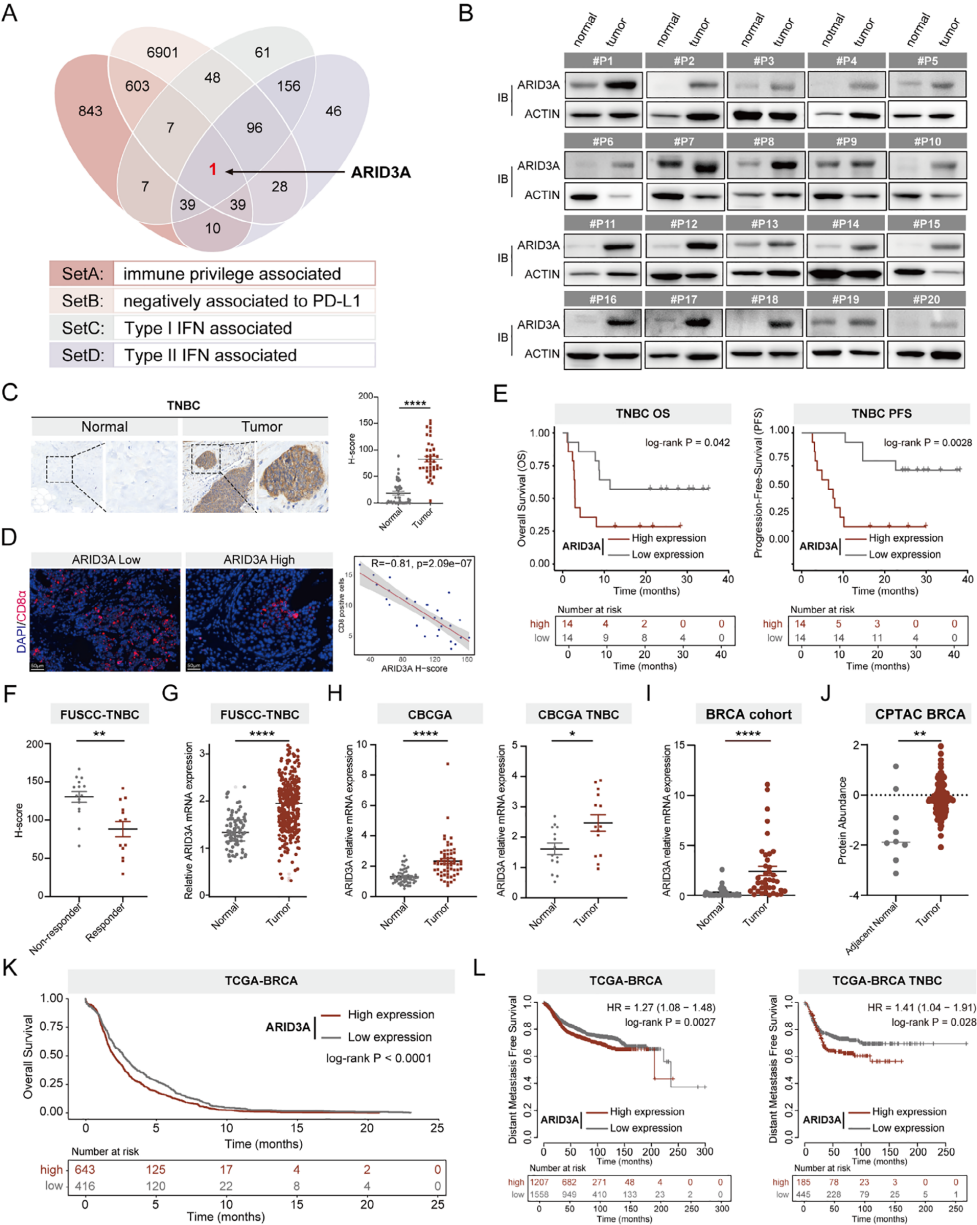
*ARID3A* is upregulated in breast cancer and is associated with immunosuppression and poor clinical outcomes. (A) Schematic workflow identifying *ARID3A* as a key candidate through integrated multi‐cohort analyses based on gene sets associated with immune privilege, negative correlation with PD‐L1, and type I/II interferon signaling. (B) Immunoblot analysis of ARID3A protein levels in tumor (T) and paired adjacent normal (N) tissues from the BRCA cohort (*n* = 20 paired samples). ACTIN served as the loading control. (C) Representative immunohistochemistry (IHC) images (left) and H‐score quantification (right) of ARID3A expression in normal breast tissues and TNBC tumors from an immunotherapy cohort. Data are presented as mean ± SEM. Statistical significance was determined by a two‐tailed unpaired Student's t‐test. (D) Multiplex immunofluorescence analysis showing an inverse correlation between ARID3A expression and CD8^+^ T cell infiltration in TNBC tumors (*n* = 28 patients). Statistical significance was determined by Pearson correlation analysis (*R* = −0.81, *p* = 2.09 × 10^−^^7^). (E) Kaplan‐Meier analysis of overall survival (OS, left) and progression‐free survival (PFS, right) in the TNBC immunotherapy cohort (*n* = 28 patients), stratified by ARID3A protein H‐scores. *P* values were calculated using the log‐rank test. (F) ARID3A IHC H‐scores in TNBC patients exhibiting divergent responses to immunotherapy (responders, *n* = 13; non‐responders, *n* = 15). Data are presented as mean ± SEM. Statistical significance was determined by a two‐tailed unpaired Student's *t*‐test. (G) *ARID3A* mRNA expression in normal versus tumor tissues from the FUSCC‐TNBC cohort. Data are presented as mean ± SEM. Statistical significance was determined by a two‐tailed unpaired Student's *t*‐test. (H) *ARID3A* mRNA expression in normal versus tumor tissues across the broader CBCGA cohort (left) and its specific TNBC subgroup (right). Data are presented as mean ± SEM. Statistical significance was determined by two‐tailed unpaired Student's *t*‐tests. (I) Quantitative PCR analysis of *ARID3A* mRNA expression in breast cancer tissues versus adjacent normal tissues in an independent BRCA cohort. Data are presented as mean ± SEM. Statistical significance was determined by a two‐tailed unpaired Student's *t*‐test. (J) ARID3A protein abundance in the CPTAC BRCA cohort. Data are presented as mean ± SEM. Statistical significance was determined by a two‐tailed unpaired Student's *t*‐test. (K, L) High *ARID3A* expression predicts poor survival in TCGA cohorts. (K) Kaplan‐Meier analysis of overall survival in the TCGA‐BRCA cohort stratified by *ARID3A* mRNA expression. (L) Kaplan‐Meier analyses of distant metastasis‐free survival (DMFS) in the full TCGA‐BRCA cohort (left) and the TNBC subgroup (right). *P* values were calculated using the log‐rank test. Statistical Analysis: Quantitative data are presented as mean ± SEM unless otherwise specified. Comparisons between two independent groups were performed using two‐tailed unpaired Student's *t*‐tests. Correlation analysis was performed using Pearson's correlation. Survival analyses were conducted using the log‐rank test. Significance is denoted as: *p < 0.05, **p < 0.01, ***p < 0.001, ****p < 0.0001. Sample sizes (*n*) represent biological replicates (individual patients or samples) and are indicated in each panel. Consistent with standard HGNC guidelines, *ARID3A* (italicized) denotes mRNA/gene transcripts, whereas ARID3A (non‐italicized) denotes the protein. All statistical analyses were performed using GraphPad Prism or R software.

Intersection of these datasets yielded a single overlapping gene: ARID3A (Figure 1A), suggesting its role as a core regulator linking immune‐privileged states to suppressed interferon‐driven immunity. Consistently, pan‐cancer analysis in the Cancer Cell Line Encyclopedia (CCLE) revealed an inverse correlation between ARID3A and PD‐L1 mRNA levels (Figure S1A), supporting its distinct immunomodulatory function.

We first validated ARID3A expression and clinical relevance in focused institutional cohorts. Western blot analysis of 20 paired BRCA samples confirmed significant ARID3A protein upregulation in tumors versus adjacent normal tissues (Figure 1B). This was corroborated in an independent immunotherapy‐treated TNBC cohort (*n* = 28) by immunohistochemistry (IHC), which showed higher ARID3A levels in TNBC than in normal breast tissue (Figure 1C), with quantitative H‐score analysis confirming patient‐level overexpression. Singleplex immunofluorescence in these TNBC samples revealed a significant inverse correlation between ARID3A expression and intratumoral CD8^+^ T cell density (Figure 1D), implicating ARID3A in immune exclusion. Clinically, high ARID3A expression was associated with significantly shorter overall survival (OS) and progression‐free survival (PFS) (Figure 1E), and non‐responders to immunotherapy exhibited higher ARID3A mRNA levels than responders (Figure 1F), directly linking *ARID3A* to treatment resistance.

We next expanded our analysis to larger clinical datasets. Examination of the FUSCC‐TNBC cohort, the comprehensive CBCGA cohort (all BRCA subtypes), and a dedicated qPCR cohort (*n* = 39) consistently demonstrated significant ARID3A mRNA upregulation (Figure 1G–I). This transcriptional signature was further validated at the protein level using the CPTAC‐BRCA dataset (Figure 1J). Additionally, *ARID3A* expression was elevated in tumors compared to normal tissues across multiple TCGA cohorts (Figure S1B,C).

We then assessed the prognostic value of ARID3A in broader breast cancer contexts. In the TCGA‐BRCA cohort, high ARID3A expression correlated with shorter OS and distant metastasis‐free survival (DMFS) in both the overall population and the TNBC subgroup (Figure 1K,L), reinforcing its role as a biomarker of immune exclusion and poor prognosis.

To explore ARID3A's pan‐cancer significance, we analyzed TCGA data across multiple malignancies. ARID3A was consistently overexpressed in tumors versus normal tissues, and its elevated expression correlated with poorer OS in several aggressive cancers, including glioblastoma (GBM), kidney renal clear cell carcinoma (KIRC), lung adenocarcinoma (LUAD), skin cutaneous melanoma (SKCM), and stomach adenocarcinoma (STAD) (Figure S1D–H), positioning it as a potential pan‐cancer prognostic marker.

Further supporting its immune‐evasive role, analysis of public single‐cell RNA‐seq data (GSE205506) showed that *ARID3A*‐high tumors display an immune‐cold microenvironment, marked by reduced infiltration of key leukocyte subsets—especially cytotoxic CD8^+^ T cells (Figure S1I–K). This immunosuppressive trait persisted across clinical stages, with ARID3A‐high tumors consistently exhibiting lower T cell infiltration at each stage (Figure S1L,M). To directly investigate how tumor‐intrinsic *ARID3A* shapes the functional state of infiltrating T cells, we performed a head‐to‐head comparison of CD8^+^ T cells from *ARID3A*‐high versus *ARID3A*‐low tumors within our scRNA‐seq dataset. This analysis, presented as a volcano plot (Figure S1N), uncovered a distinct transcriptional signature associated with *ARID3A* status: in *ARID3A*‐low tumors, effector molecules such as granzyme B (*GZMB*) and *IFNG* were significantly upregulated, while exhaustion markers were downregulated, supporting the notion that ARID3A levels in tumors influence CD8^+^ T cell functionality. Finally, in a neoadjuvant anti‐PD‐1 colorectal cancer cohort, pathological complete responders (pCR) had lower *ARID3A* expression than non‐pCR cases (Figure S1O), indicating that *ARID3A* is associated with primary checkpoint blockade resistance beyond breast cancer.

In summary, our multi‐cohort, multi‐platform analysis establishes ARID3A as a consistently overexpressed tumor antigen linked to immune exclusion, therapy resistance, and adverse clinical outcomes. These findings nominate ARID3A as a candidate biomarker for risk stratification and a potential therapeutic target for reversing the immune‐cold tumor phenotype.

### 2.2 ARID3A Deficiency Sensitizes Tumor Cells to T‐Cell‐Mediated Cytotoxicity

Based on our finding that ARID3A overexpression correlates with an immunosuppressive phenotype (Figure 1), we sought to define its functional role in tumor‐immune interactions. Given the rarity of inactivating *ARID3A* mutations across cancers (Figure S2A), we focused on its gain‐of‐function effects. We generated *Arid3a*‐deficient murine breast cancer cells (EO771 and 4T1), with immunoblotting confirming efficient protein ablation (Figure S2B).

**FIGURE 2:**
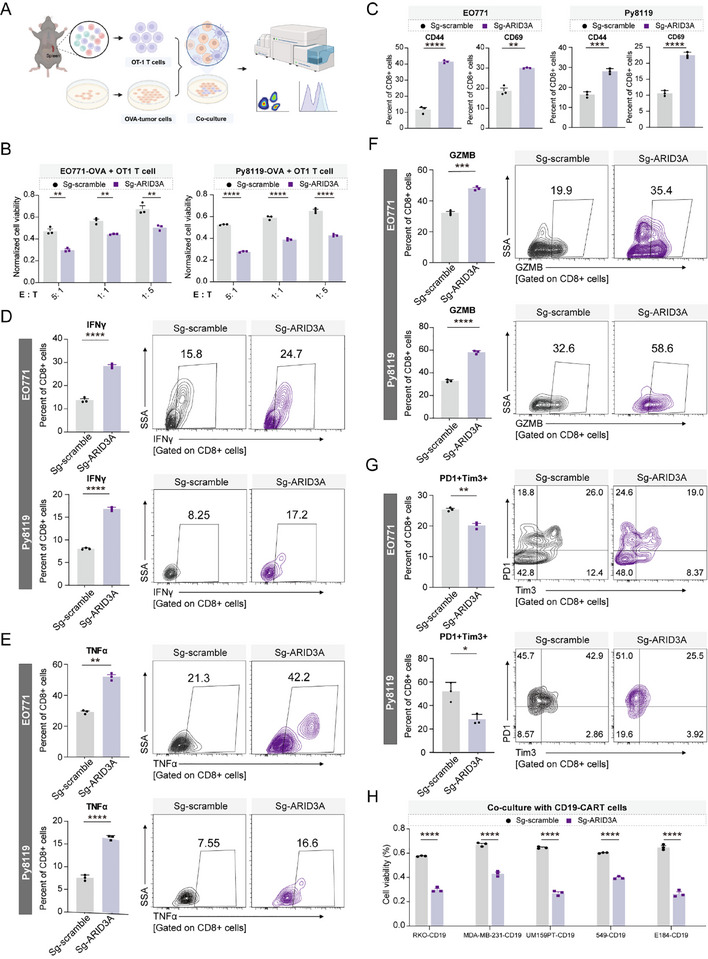
*Arid3a* suppresses tumor cell susceptibility to T cell‐mediated cytotoxicity. (A) Schematic illustration of the OT‐I CD8^+^ T cell co‐culture assay with OVA‐expressing tumor cells. Created with BioRender.com (Agreement No. IX28WVJ76S). (B) *Arid3a* deficiency enhances OT‐I CD8^+^ T cell‐mediated cytotoxicity. Viability of OVA‐expressing EO771 (left) and Py8119 (right) tumor cells following a 24‐h co‐culture with OT‐I CD8^+^ T cells at the indicated effector‐to‐target (E:T) ratios (*n* = 3 biologically independent experiments). (C) *Arid3a* deficiency promotes OT‐I CD8^+^ T cell activation. Quantification of CD44^+^ and CD69^+^ OT‐I CD8^+^ T cells following a 12‐h co‐culture with sg‐scramble‐ or sg‐*Arid3a*‐expressing EO771‐OVA (left) and Py8119‐OVA (right) tumor cells (*n* = 3 biologically independent experiments). (D–F) *Arid3a* deficiency enhances OT‐I CD8^+^ T cell effector function. Flow cytometry analysis and quantification of intracellular (D) IFN‐γ, (E) TNF‐α, and (F) granzyme B (GZMB) in OT‐I CD8^+^ T cells after co‐culture with sg‐scramble‐ or sg‐*Arid3a*‐expressing EO771‐OVA (top panels) and Py8119‐OVA (bottom panels) cells (*n* = 3 biologically independent experiments). (G) *Arid3a* deficiency reduces OT‐I CD8^+^ T cell exhaustion. Flow cytometry analysis and quantification of PD‐1 and TIM‐3 co‐expression on OT‐I CD8^+^ T cells after a 24‐h co‐culture with sg‐scramble‐ or sg‐*Arid3a*‐expressing EO771‐OVA and Py8119‐OVA cells (*n* = 3 biologically independent experiments). (H) *ARID3A* deficiency sensitizes diverse CD19^+^ solid tumor cells to CD19‐CAR T cell‐mediated killing. Viability of five CD19‐engineered solid tumor cell lines (RKO, MDA‐MB‐231, SUM159PT, A549, and EFE184) after a 24‐h co‐culture with CD19‐CAR T cells at an E:T ratio of 5:1 (*n* = 3 biologically independent experiments). Statistical Analysis: All quantitative data are presented as mean ± SEM. Statistical comparisons between two independent groups (C, D, E, F, G) were performed using two‐tailed unpaired Student's *t*‐tests. Comparisons involving two independent variables (B, H) were analyzed using two‐way ANOVA followed by Bonferroni's post‐hoc test. Significance is denoted as: *p < 0.05, **p < 0.01, ***p < 0.001, ****p < 0.0001. Sample sizes (*n*) representing biologically independent experiments are explicitly indicated for each panel. For visual clarity in figures, mouse gene symbols used as labels (e.g., Sg‐ARID3A) are presented in uppercase, while they refer to the corresponding mouse genes (e.g., *Arid3a*) according to MGI guidelines. All statistical analyses were performed using GraphPad Prism software.

To test if ARID3A modulates tumor susceptibility to T cells, we employed an antigen‐specific co‐culture system with OT‐I CD8^+^ T cells and OVA‐expressing tumor targets. Genetic ablation of *Arid3a* in EO771‐OVA and Py8119‐OVA cells, both of which are derived from C57BL/6 mice and therefore MHC‐matched (H‐2Kb) to OT‐I T cells, significantly enhanced their killing by OT‐I T cells across multiple effector‐to‐target (E:T) ratios (Figure 2A,B). This effect was not attributable to altered intrinsic proliferation, apoptosis, or migration of the *Arid3a* deficient cells (Figure S2C–E). The enhanced cytotoxicity was generalizable, as *Arid3a* deficiency also sensitized other OVA^+^ syngeneic tumor lines (B16‐F10, MC38, LLC) to OT‐I T cell killing (Figure S2F). Corroborating these functional data, tumors with low *ARID3A* expression exhibited significantly higher immune cytotoxicity scores in multiple TCGA datasets (Figure S2G).

We next investigated whether increased killing reflected enhanced T cell activation. Flow cytometric analysis revealed that OT‐I cells co‐cultured with ARID3A‐KD targets exhibited elevated expression of early (CD69) and late (CD44) activation markers, along with increased frequencies of IFN‐γ^+^ and TNF‐α^+^ producers (Figure 2C–E). This heightened effector response was further evidenced by enhanced GZMB induction (Figure 2F). Notably, this potentiated anti‐tumor function was not accompanied by upregulated exhaustion markers, as PD‐1 and TIM‐3 levels remained unchanged (Figure 2G). Thus, tumor‐intrinsic ARID3A limits the magnitude of T cell activation and effector differentiation, without promoting T cell exhaustion.

To assess the translational generality of this mechanism, we extended our analysis to human tumors targeted by chimeric antigen receptor (CAR) T cells. *ARID3A* deficiency consistently sensitized a panel of five CD19^+^ human tumor lines to killing by autologous CD19‐directed CAR T cells (Figure 2H), confirming that ARID3A's protective role transcends antigen specificity, species, and tumor lineage.

In summary, these results establish ARID3A as a tumor‐intrinsic regulator of T cell‐mediated cytotoxicity. By constraining T cell activation and cytolytic programming, ARID3A effectively shields tumor cells from both endogenous and engineered cytotoxic lymphocytes, establishing a novel mechanism of tumor‐intrinsic immune evasion.

### 2.3 ARID3A Deficiency Restricts Tumor Growth in Immunocompetent Hosts by Enhancing CD8^+^ T Cell Immunity

To determine whether ARID3A promotes tumor growth primarily through immunomodulation rather than intrinsic proliferation, we compared the growth of *Arid3a*‐deficient tumors in immunodeficient versus immunocompetent mice.

**FIGURE 3:**
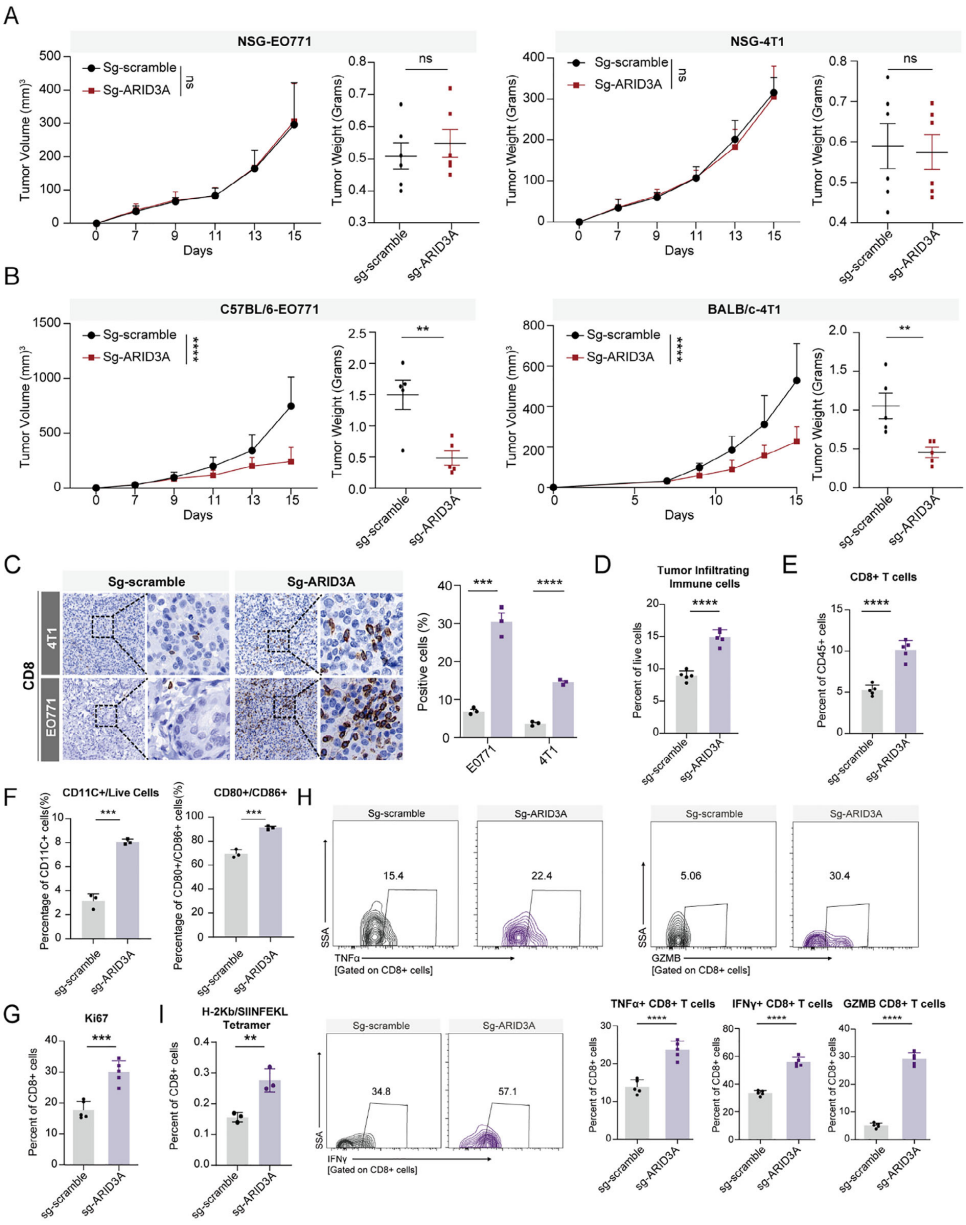
*Arid3a* promotes tumor growth and suppresses anti‐tumor T cell immunity in vivo. (A) *Arid3a* deficiency does not affect intrinsic tumor growth in the absence of adaptive immunity. Tumor growth curves (left panels) and final tumor weights (right panels) in immunodeficient NSG mice xenografted with sg‐scramble‐ or sg‐*Arid3a*‐expressing EO771 and 4T1 cells (*n* = 6 mice per group). (B) *Arid3a* deficiency significantly inhibits tumor growth in immunocompetent hosts. Tumor growth curves (left panels) and final tumor weights (right panels) of syngeneic EO771 tumors in C57BL/6 mice and 4T1 tumors in BALB/c mice (*n* = 5 mice per group). (C) *Arid3a* deficiency enhances CD8^+^ T cell infiltration. Representative IHC images (left) and quantification (right) of CD8^+^ T cells in EO771 and 4T1 tumors (*n* = 3 biologically independent samples). Scale bars as indicated. (D, E) Flow cytometry quantification of (D) total tumor‐infiltrating immune cells (CD45^+^) and (E) CD8^+^ T cells as a percentage of CD45^+^ leukocytes in sg‐scramble‐ and sg‐*Arid3a*‐EO771 tumors (*n* = 5 tumors per group). (F) *Arid3a* deficiency enhances the infiltration and activation of DC‐enriched populations in vivo. Flow cytometry quantification of the percentage of CD45^+^CD11c^+^ cells among total live cells (left) and activated (CD80^+^CD86^+^) cells within the CD11c^+^ population (right) (*n* = 3 tumors per group). (G) *Arid3a* deficiency promotes CD8^+^ T cell proliferation. Flow cytometry quantification of Ki‐67^+^ cells among tumor‐infiltrating CD8^+^ T cells (*n* = 5 tumors per group). (H) *Arid3a* deficiency enhances CD8^+^ T cell effector function in vivo. Representative flow cytometry contour plots (top and bottom left) and quantification (bottom right) of intracellular TNF‐α, IFN‐γ, and granzyme B (GZMB) production in tumor‐infiltrating CD8^+^ T cells (*n* = 5 tumors per group). (I) MHC tetramer staining demonstrates enhanced antigen‐specific CD8^+^ T‐cell responses in vivo. Quantification of SIINFEKL/H‐2K^b^ tetramer‐positive cells among tumor‐infiltrating CD8^+^ T cells from OVA‐expressing EO771 tumors (*n* = 3 tumors per group). Statistical Analysis: All quantitative data are presented as mean ± SEM. Comparisons between two independent groups (bar graphs and final tumor weights) were performed using two‐tailed unpaired Student's *t*‐tests. Tumor growth curves were analyzed using repeated‐measures two‐way ANOVA followed by Bonferroni's post hoc test. Significance is denoted as: *p < 0.05, **p < 0.01, ***p < 0.001, ****p < 0.0001; ns, not significant. Sample sizes (*n*) represent biological replicates (individual mice or tumors) per group, corresponding strictly to the individual data points shown. For visual clarity in figures, mouse gene symbols used as labels (e.g., Sg‐ARID3A, sgArid3a) are presented with varied capitalization, while they refer to the corresponding mouse genes (e.g., *Arid3a*) according to MGI guidelines. All statistical analyses were performed using GraphPad Prism software.

We first assessed tumor growth in NSG immunodeficient mice, where control and *Arid3a*‐deficient tumors exhibited comparable growth kinetics and final weights (Figure 3A), indicating that ARID3A does not affect cell‐autonomous proliferation in the absence of adaptive immunity. In striking contrast, *Arid3a* deficiency profoundly suppressed tumor progression in syngeneic immunocompetent hosts‐C57BL/6 mice bearing EO771 tumors and BALB/c mice bearing 4T1 tumors‐significantly reducing both tumor volume and endpoint mass (Figure 3B). These results establish that the pro‐tumorigenic function of ARID3A is strictly dependent on an intact immune system.

We next profiled the tumor immune landscape. IHC revealed a marked increase in intra‐tumoral CD8^+^ T cell infiltration in *Arid3a*‐deficient EO771 lesions, with representative images and quantification across biological replicates (Figure 3C). Flow cytometric analyses confirmed these findings: total leukocyte (CD45^+^) infiltration increased, and the proportion of CD8^+^ T cells rose in *Arid3a*‐deficient tumors relative to controls (Figure 3D,E). Concomitantly, the proportion of tumor‐infiltrating DC‐enriched populations (CD45^+^CD11c^+^) among total live cells was significantly elevated (approximately 8% vs. 3% in controls). Furthermore, these recruited cells exhibited a highly activated phenotype, characterized by increased surface co‐expression of the co‐stimulatory molecules CD80 and CD86 (∼90% vs. ∼68% in controls) (Figure 3F). Beyond modulating CD8^+^ T cell abundance, ARID3A further restricts CD8^+^ T cell functional fitness in situ. Loss of *Arid3a* enhanced CD8^+^ T cell proliferation—as evidenced by elevated Ki‐67 expression (Figure 3G)—and boosted effector programming: intracellular staining revealed increased frequencies of IFN‐γ^+^, TNF‐α^+^, and GZMB^+^ CD8^+^ T cells within *Arid3a*‐deficient tumors (Figure 3H). This coordinated augmentation of proliferative and cytolytic states, together with enhanced CD8^+^ T cell infiltration, explains the immune‐dependent tumor control observed in immunocompetent hosts.

To directly quantify antigen specificity in vivo, we performed SIINFEKL/H‐2Kb tetramer staining in the EO771‐OVA syngeneic model. *Arid3a* deficiency significantly increased the frequency of tetramer‐positive CD8+ T cells by approximately 1.9‐fold (from 0.14% to 0.26%), providing direct evidence that *Arid3a* loss expands tumor antigen‐specific CTL responses within the tumor microenvironment. (Figure 3I).

To contextualize these mechanistic insights clinically, we integrated data from ICB cohorts. Using the Tumor Immune Dysfunction and Exclusion (TIDE) algorithm—a validated predictor of ICB response [24]—we observed significantly higher *ARID3A* expression in predicted non‐responders relative to predicted responders across multiple cancer types (Figure S3A). Consistently, analysis of independent ICB‐treated datasets revealed that elevated *ARID3A* expression was associated with inferior survival and enriched among non‐responders, whereas predicted or observed responders were characterized by lower *ARID3A* expression [25] (Figure S3B,C). Kaplan–Meier analyses of ICB‐treated patient cohorts further supported these associations: low *ARID3A* expression was associated with significantly prolonged overall and progression‐free survival following anti‐PD‐1 or anti‐PD‐L1 therapy (Figure S3D) [26].

Spatial transcriptomic analysis of anti–PD‐1–treated hepatocellular carcinoma samples (*n* = 15 tumors) [27, 28] revealed that *ARID3A* expression was higher in non‐responders and diffusely distributed throughout the tumor parenchyma (Figure S3E). These clinical observations mirror the immune‐excluded, low‐cytotoxicity phenotype driven by *Arid3a* in our murine models, supporting its potential role as a biomarker of primary resistance to PD‐1–based immunotherapy.

Together, these findings establish ARID3A as a tumor‐intrinsic suppressor of antigen‐specific and effector CD8^+^ T‐cell immunity. While dispensable for growth in immunodeficient settings, ARID3A is critical for maintaining an immune‐cold tumor microenvironment in immunocompetent hosts.

Having established that ARID3A restrains anti‐tumor immunity in vivo, we next sought to define the molecular mechanism through which it suppresses immune activation within the tumor microenvironment.

### 2.4 ARID3A Represses Cytosolic Nucleic Acid Sensing and Type I Interferon Signaling

**FIGURE 4:**
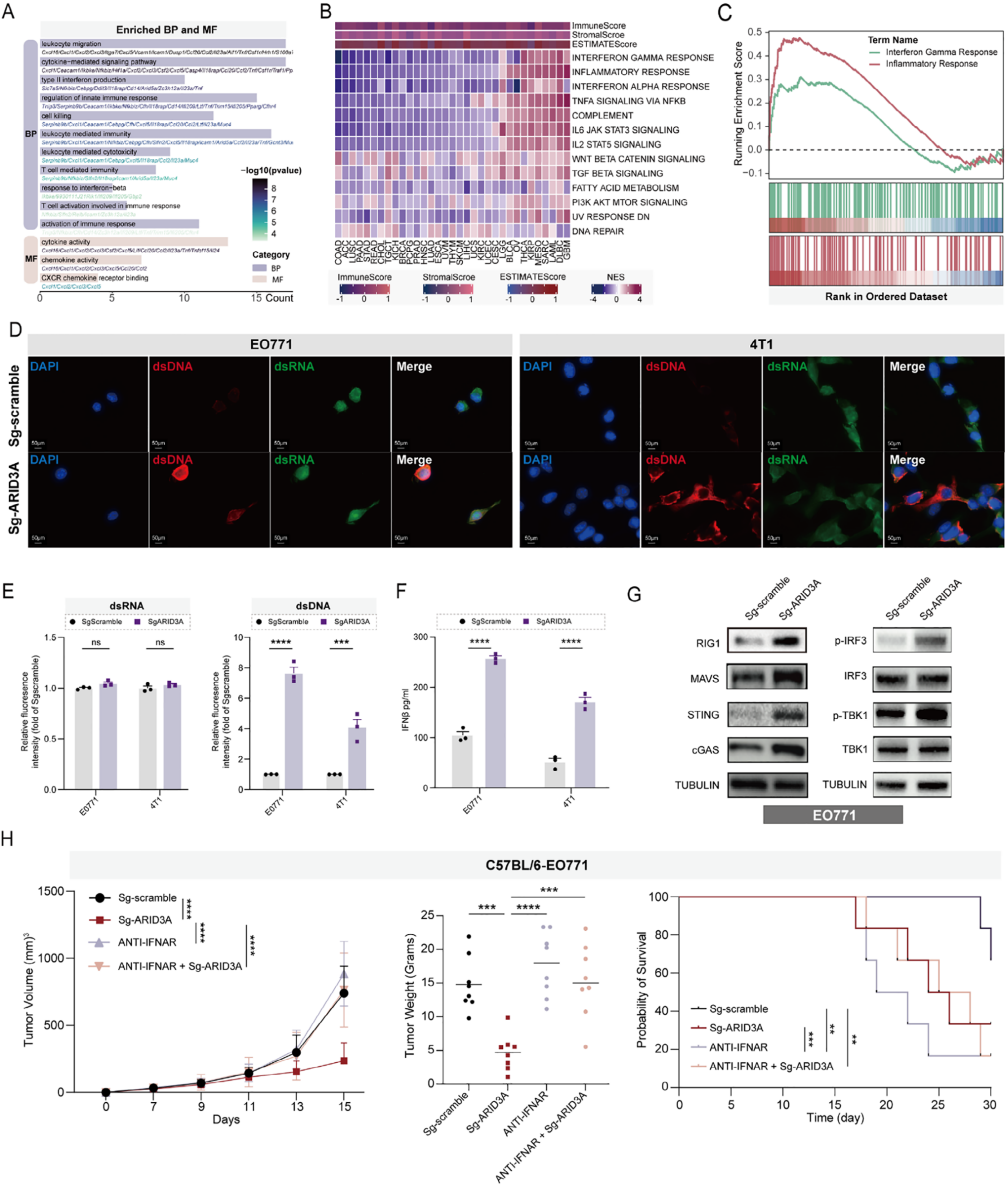
*Arid3a* represses innate immune signaling by inhibiting cytosolic nucleic acid sensing and type I interferon production. (A) Gene Ontology (GO) enrichment analysis of upregulated genes in sg‐*Arid3a* EO771 cells reveals activation of immune‐related biological processes (BP) and molecular functions (MF) based on RNA sequencing data (*n* = 3 biologically independent samples). (B) Heatmap showing enrichment scores of Hallmark gene sets and ESTIMATE‐derived stromal and immune scores in TCGA human pan‐cancer cohorts stratified by *ARID3A* expression. (C) Gene set enrichment analysis (GSEA) demonstrating significant enrichment of the Interferon Gamma Response and Inflammatory Response gene sets in *Arid3a*‐deficient cells. (D, E) *Arid3a* deficiency induces the accumulation of cytosolic nucleic acids. (D) Representative confocal immunofluorescence images showing nuclear DNA (DAPI, blue), cytosolic double‐stranded DNA (dsDNA, red), and double‐stranded RNA (dsRNA, green) in sg‐scramble‐ or sg‐*Arid3a*‐expressing EO771 and 4T1 cells. All images were acquired using identical acquisition settings and processed in parallel. Scale bars = 50 µm. (E) Quantification of relative cytosolic dsDNA and dsRNA immunofluorescence signals (*n* = 3 biologically independent experiments). (F) *Arid3a* deficiency increases IFN‐β secretion. Enzyme‐linked immunosorbent assay (ELISA) measurement of IFN‐β levels in the supernatants of sg‐scramble‐ or sg‐*Arid3a*‐expressing EO771 and 4T1 cells (*n* = 3 biologically independent experiments). (G) *Arid3a *deficiency upregulates innate immune signaling proteins. Representative immunoblot analysis of RIG‐I, MAVS, STING, and cGAS (left panel), and phosphorylated IRF3 (p‐IRF3), total IRF3, phosphorylated TBK1 (p‐TBK1), and total TBK1 (right panel) in sg‐scramble‐ or sg‐*Arid3a*‐expressing EO771 cells. Tubulin served as the loading control. (H) Blocking type I interferon signaling reverses the tumor‐suppressive effect of *Arid3a* deficiency in vivo. C57BL/6 mice bearing sg‐scramble‐ or sg‐*Arid3a*‐EO771 tumors were treated with an anti‐IFNAR1 antibody or isotype control. Left, tumor growth curves; middle, final tumor weights; right, Kaplan‐Meier survival analysis (*n* = 6 mice per group). Statistical Analysis: All quantitative data are presented as mean ± SEM. Statistical comparisons between two independent groups (E, F) were performed using two‐tailed unpaired Student's *t*‐tests. Comparisons among multiple groups (H, middle panel) were performed using one‐way ANOVA with Bonferroni's post‐hoc correction. Tumor growth curves (H, left panel) were analyzed using repeated‐measures two‐way ANOVA followed by Bonferroni's post hoc test. Survival analyses (H, right panel) were conducted using the log‐rank test. Significance is denoted as: *p < 0.05, **p < 0.01, ***p < 0.001, ****p < 0.0001; ns, not significant. Sample sizes (*n*) represent biologically independent samples or animals, as indicated. All statistical analyses were performed using GraphPad Prism software.

To elucidate the mechanistic link between ARID3A and the observed immune phenotypes, we first characterized the transcriptional alterations induced by *Arid3a* deficiency. RNA sequencing of sg‐*Arid3a* EO771 cells followed by Gene Ontology (GO) enrichment analysis revealed a robust upregulation of genes involved in immune‐related processes, most notably the regulation of innate immune responses, along with leukocyte migration, cytokine‐mediated signaling, and cytokine activity (Figure 4A).

Using TCGA pan‐cancer data, Hallmark pathway and ESTIMATE analyses showed that tumors with low *ARID3A* expression exhibited increased enrichment for immune‐related pathways and significantly elevated stromal/immune scores (Figure 4B)—indicative of a tumor microenvironment permissive to leukocyte infiltration. To directly test this association, Gene Set Enrichment Analysis (GSEA) confirmed significant enrichment of Interferon Gamma Response and Inflammatory Response gene sets—the most prominent enriched immune programs—in *Arid3a*‐deficient cells (Figure 4C).

We next investigated endogenous danger‐sensing pathways, using a schematic [29] outlining dsDNA and dsRNA sensing cascades that drive IRF3 activation and IFN‐I induction (Figure S4A). Confocal microscopy and quantitative immunofluorescence revealed marked cytosolic accumulation of double‐stranded DNA (dsDNA) in *Arid3a*‐deficient cells, with minimal changes in double‐stranded RNA (dsRNA), accompanied by elevated IFN‐β secretion (ELISA; Figure 4D–F). Immunoblotting showed activation of canonical nucleic acid sensors: cGAS and STING (the core dsDNA‐sensing axis) were strongly activated, while RIG‐I and MAVS (the dsRNA‐sensing axis) showed modest activation—consistent with minimal dsRNA changes. Downstream effectors also exhibited increased phosphorylation of IRF3 and TBK1 (total protein levels were unchanged). These findings position ARID3A upstream of the type I interferon signaling module (Figure 4G, Figure S4E).

*Arid3a* deficiency also suppressed DNA‐repair pathways and increased DNA damage, evident from elevated γH2AX levels—providing a plausible source for cytosolic DNA leakage (Figure S4B–D). At the transcriptional level, *Arid3a*‐deficient cells and tumors had upregulated *Ifnb1*, along with interferon‐stimulated and T cell‐supportive genes (*Cxcl9, Cxcl10, Usp18, Igtp, Oas1g, Tap1, and Isg15*; Figure S4F–H). Notably, IFI200 family components (*Ifi209, Ifi205*)—known to enhance dsDNA sensing upstream of cGAS‐STING—were induced, as was the kinase *Ikbke*, which amplifies RIG‐I‐MAVS signaling downstream of dsRNA (Figure S4I–J). These transcriptional changes likely reinforce activation of both nucleic acid‐sensing axes, even with minimal dsRNA accumulation.

Finally, to establish the functional relevance of type I IFN signaling in vivo, we blocked IFNAR1 in syngeneic hosts bearing control or *Arid3a*‐deficient tumors. IFNAR1 inhibition abrogated the growth suppression caused by *Arid3a* deficiency, restoring tumor kinetics and final mass to control levels (Figure 4H). This rescue experiment establishes type I interferon signaling as a necessary downstream mediator required for the tumor suppression induced by *Arid3a* deficiency.

Together, these findings demonstrate that ARID3A functions as a key repressor of endogenous nucleic acid sensing and type I interferon production. By restraining this axis, ARID3A suppresses the expression of critical chemokines and interferon‐stimulated genes that are essential for recruiting and potentiating CD8^+^ T cell activity within the tumor microenvironment.

Given that ARID3A‐mediated suppression of the type I interferon pathway represents a tractable immune‐evasion mechanism, we reasoned that pharmacologically targeting ARID3A could reactivate this innate immune axis and restore tumor immunogenicity. We therefore performed a structure‐based drug screen to identify compounds capable of directly engaging ARID3A.

### 2.5 Rimegepant Targets ARID3A and Activates Innate Immune Signaling

**FIGURE 5:**
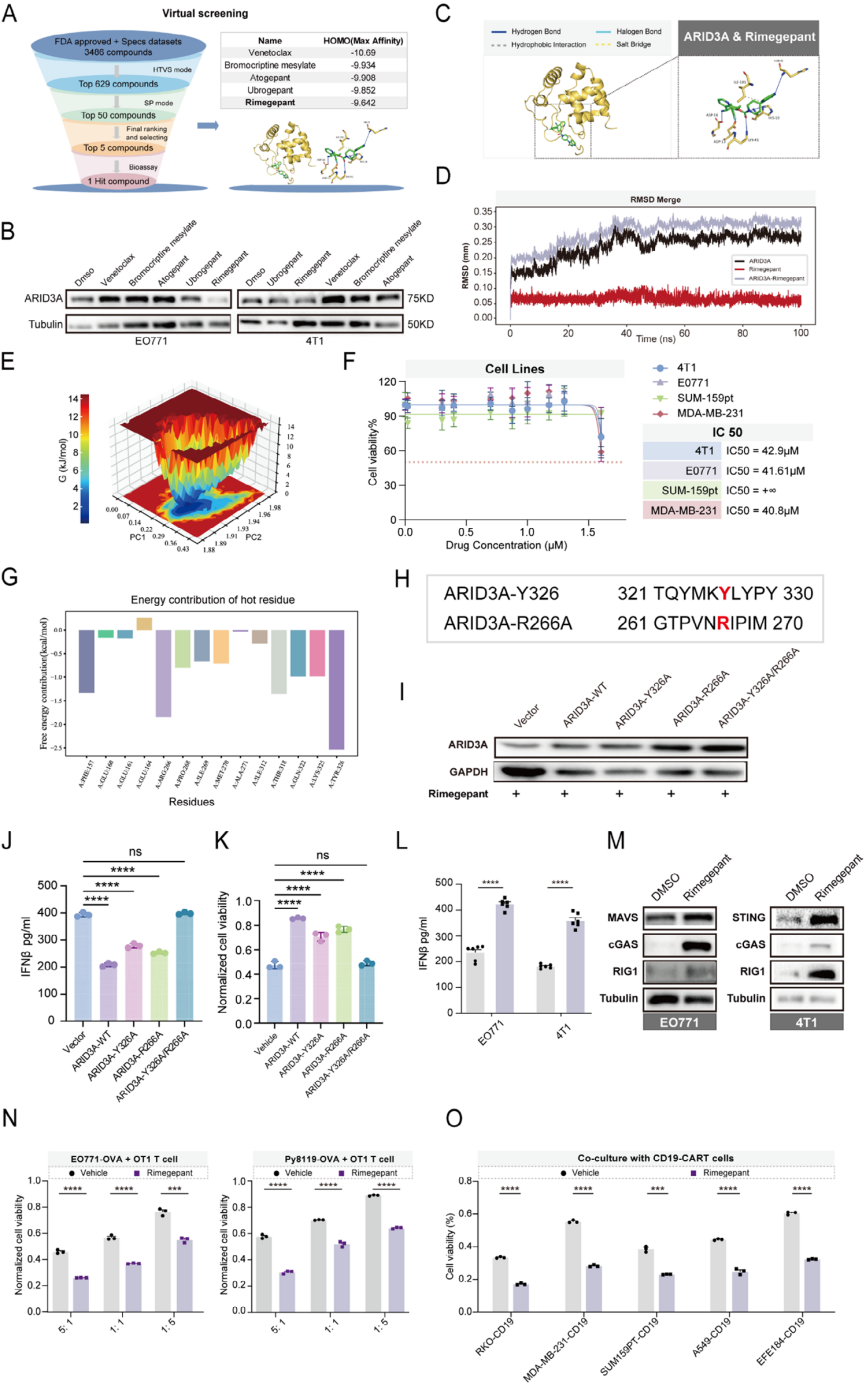
Rimegepant targets ARID3A and activates innate immune signaling to sensitize tumors to T cell–mediated cytotoxicity. (A) Schematic workflow of virtual screening to identify ARID3A‐targeting compounds from FDA‐approved and Specs compound libraries. (B) Immunoblot analysis identifying rimegepant as a top candidate that reduces ARID3A protein levels in EO771 and 4T1 cells. Tubulin served as the loading control. (C) Molecular docking model predicting the binding pose and detailed interactions between rimegepant (green) and the ARID3A pocket (gold). (D) Molecular dynamics (MD) simulation showing the structural stability of the ARID3A–rimegepant complex, as assessed by root‐mean‐square deviation (RMSD) over a 100‐ns simulation. (E) Free energy landscape (FEL) analysis illustrating ARID3A conformational states in the presence of rimegepant, plotted as principal components (PC1 vs. PC2). (F) Dose‐response curves and half‐maximal inhibitory concentration (IC_50_) values of rimegepant in the indicated breast cancer cell lines. (G) Per‐residue free energy decomposition identifying the major energy‐contributing residues (including R266 and Y326) at the predicted binding interface. (H) Schematic illustrating the site‐directed single mutations (R266A and Y326A) generated within the ARID3A binding pocket. (I) Immunoblot analysis of ARID3A levels in ARID3A‐deficient cells rescued with vector, wild‐type (WT) ARID3A, single mutants (Y326A, R266A), or the double mutant (Y326A/R266A) following rimegepant treatment (+). GAPDH served as a loading control. (J) IFN‐β secretion levels measured by ELISA in the indicated ARID3A‐rescued cell lines following rimegepant treatment (*n* = 3 biologically independent experiments). Statistical significance was determined by one‐way ANOVA followed by Bonferroni's post hoc test. (K) Normalized cell viability in the indicated ARID3A‐rescued cell lines following rimegepant treatment (*n* = 3 biologically independent experiments). Statistical significance was determined by one‐way ANOVA followed by Bonferroni's post hoc test. (L) Rimegepant induces IFN‐β secretion in WT EO771 and 4T1 cells. IFN‐β levels in culture supernatants were measured by ELISA (*n* = 3 biologically independent experiments). Statistical significance was determined by a two‐tailed unpaired Student's *t*‐test. (M) Representative immunoblot analysis of key innate immune signaling components (MAVS, cGAS, RIG‐I, and STING) in EO771 and 4T1 cells treated with DMSO or rimegepant. Tubulin served as the loading control. (N) Rimegepant pretreatment enhances OT‐I CD8^+^ T cell–mediated cytotoxicity against EO771‐OVA and Py8119‐OVA tumor cells after co‐culture at indicated effector‐to‐target (E:T) ratios (*n* = 3 biologically independent experiments). Statistical significance was determined by two‐way ANOVA followed by Bonferroni's post hoc test. (O) Rimegepant sensitizes a panel of CD19^+^ human tumor cells to CD19‐CAR T cell–mediated killing (*n* = 3 biologically independent experiments). Statistical significance was determined by two‐way ANOVA followed by Bonferroni's post hoc test. Data presentation: All quantitative data are presented as mean ± SEM. Statistical analysis: Comparisons between two groups were performed using two‐tailed unpaired Student's t‐tests. Comparisons involving multiple groups or conditions were performed using one‐way or two‐way ANOVA followed by Bonferroni's post hoc test, as indicated. Significance notation: *p < 0.05, **p < 0.01, ***p < 0.001, ****p < 0.0001, ns = not significant. Sample size (*n*): *n* represents biologically independent experiments unless otherwise indicated. Software: Statistical analyses were performed using GraphPad Prism.

To identify small molecules targeting ARID3A, we performed virtual screening and selected five FDA‐approved candidates for experimental validation (Figure 5A and Figure S5A). Immunoblotting in EO771 and 4T1 cells revealed that rimegepant most effectively reduced ARID3A protein levels, establishing it as the lead compound (Figure 5B).

Direct interaction between rimegepant and ARID3A was supported by molecular docking and molecular dynamics simulations (Figure 5C–E). Consistently, rimegepant exhibited cytotoxic activity across multiple breast cancer cell lines, with IC_50_ values ranging from 40.8 to 42.9 µM (Figure 5F).

Residue‐level free energy decomposition identified R266 and Y326 as major contributors to the predicted binding interface (Figure 5G). To experimentally validate this interaction, we generated single (R266A and Y326A) and double (Y326A/R266A) ARID3A mutants in matched‐expression rescue systems (Figure 5H). Immunoblotting demonstrated that, while wild‐type ARID3A remained susceptible to rimegepant‐induced degradation, the Y326A/R266A double mutant exhibited strong resistance (Figure 5I).

Functionally, rimegepant retained its full activity in wild‐type ARID3A‐rescued cells, whereas its effects were progressively attenuated in single‐mutant cells. Notably, the Y326A/R266A double mutation almost completely abolished rimegepant‐induced phenotypes, restoring IFN‐β secretion and cell viability to levels comparable to vector controls (Figure 5J–K). These findings provide strong genetic evidence that this binding pocket is essential for mediating the functional effects of rimegepant.

We next examined whether pharmacological targeting of ARID3A phenocopies genetic *Arid3a* deficiency. Across multiple breast cancer cell lines, rimegepant treatment induced a robust increase in IFN‐β secretion (Figure 5L). Immunoblot analysis further revealed coordinated upregulation of key innate immune sensors, including RIG‐I, MAVS, cGAS, and STING, consistent with activation of innate immune signaling pathways previously observed upon *Arid3a* depletion (Figure 5M).

To determine whether rimegepant enhances tumor cell susceptibility to T cell‐mediated killing, we pretreated OVA‐expressing murine tumor cells (EO771‐OVA and Py8119‐OVA) prior to co‐culture with OT‐I CD8^+^ T cells. Rimegepant significantly enhanced T cell cytotoxicity across multiple effector‐to‐target ratios (Figure 5N). Similarly, rimegepant pretreatment sensitized a panel of human CD19^+^ tumor cell lines to killing by CD19‐directed CAR T cells, indicating that this effect is not restricted to a specific antigen system or species (Figure 5O).

Collectively, these data demonstrate that rimegepant engages ARID3A through a defined binding interface and that this interaction is required for its immunomodulatory effects. These findings support ARID3A as a major functional target of rimegepant, although we cannot fully exclude the potential contribution of ARID3A‐independent mechanisms.

### 2.6 Rimegepant Remodels the Tumor Immune Microenvironment and Suppresses Tumor Growth In Vivo

**FIGURE 6:**
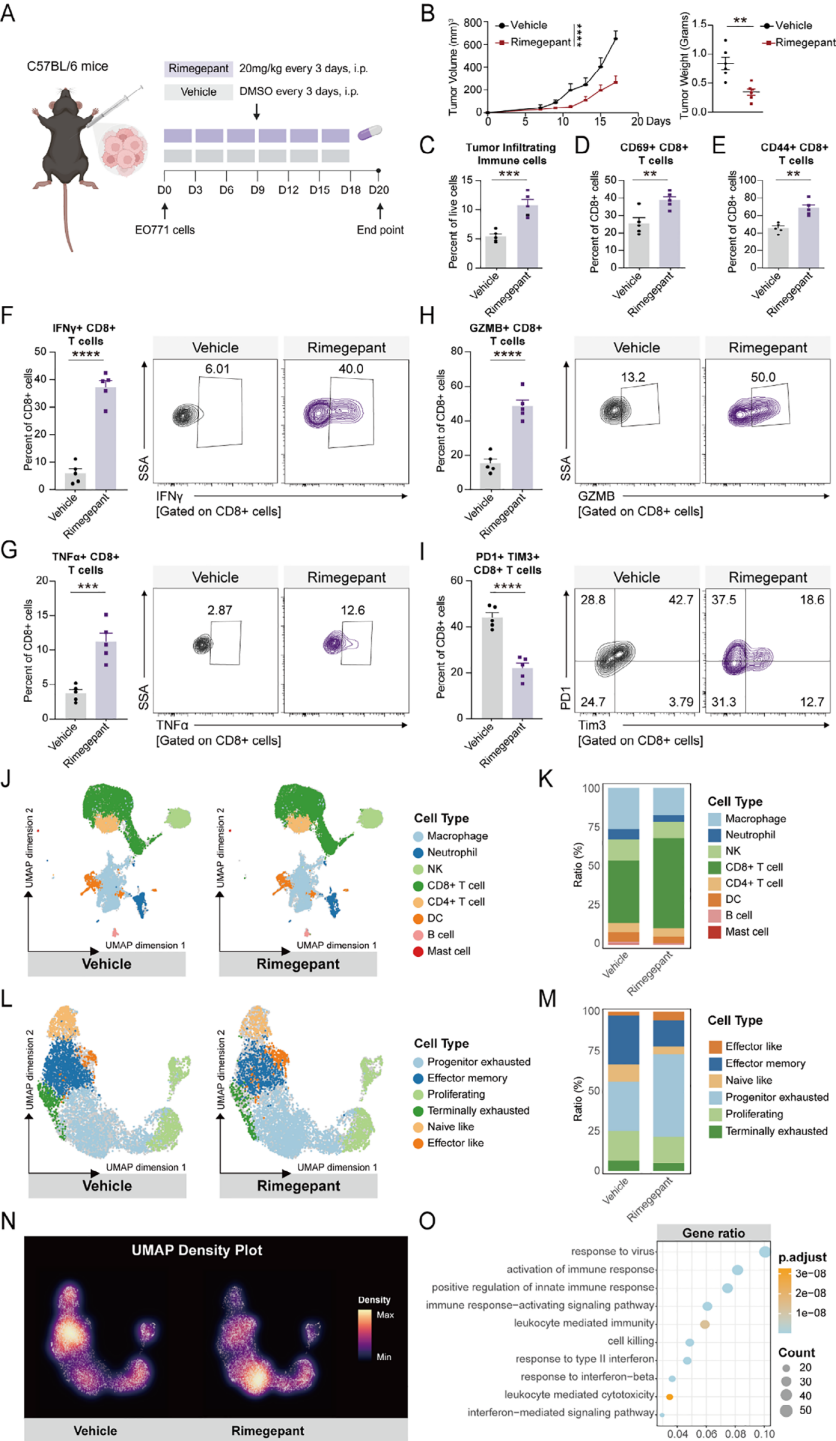
Rimegepant inhibits tumor growth and remodels the tumor immune microenvironment in vivo. (A) Schematic illustration of the in vivo treatment regimen. C57BL/6 mice bearing EO771 tumors were treated with vehicle control (DMSO) or Rimegepant (20 mg/kg) via intraperitoneal (i.p.) injection every 3 days. Created with BioRender.com (Agreement No. LR28WVJ790). (B) Rimegepant suppresses tumor growth in vivo. Tumor volume kinetics (left) and final tumor weights (right) of EO771 tumors (*n* = 5 mice per group). (C–E) Rimegepant increases T cell infiltration and activation. Flow cytometry analysis and quantification of (C) total tumor‐infiltrating immune cells (CD45^+^), (D) CD69^+^ activated CD8^+^ T cells, and (E) CD44^+^ memory/effector CD8^+^ T cells in vehicle‐ or Rimegepant‐treated tumors (*n* = 5 tumors per group). (F–H) Rimegepant enhances CD8^+^ T cell effector function. Representative flow cytometry plots and quantification of intracellular (F) IFN‐γ^+^, (G) TNF‐α^+^, and (H) granzyme B (GZMB)^+^ CD8^+^ T cells from the tumor microenvironment (*n* = 5 tumors per group). (I) Rimegepant reduces T cell exhaustion. Flow cytometry analysis of PD‐1 and TIM‐3 co‐expression on tumor‐infiltrating CD8^+^ T cells (*n* = 5 tumors per group). (J, K) Single‐cell RNA sequencing (scRNA‐seq) landscape. (J) Uniform Manifold Approximation and Projection (UMAP) visualization of tumor‐infiltrating immune cells from vehicle‐ and Rimegepant‐treated tumors. (K) Relative proportions of major immune cell populations identified by scRNA‐seq. (L, M) scRNA‐seq reveals CD8^+^ T cell subset transitions. (L) UMAP visualization and (M) relative distribution of distinct CD8^+^ T cell functional subsets in vehicle‐ or Rimegepant‐treated tumors. (N) UMAP density plots highlighting the immune cell transcriptomic redistribution following Rimegepant treatment. (O) Gene Ontology (GO) biological process enrichment analysis of differentially expressed genes identified by scRNA‐seq, revealing significant enrichment in immune activation and leukocyte‐mediated cytotoxicity pathways. Statistical Analysis: Quantitative data are presented as mean ± SEM. Comparisons between two independent groups (B right panel, C–I) were analyzed using two‐tailed unpaired Student's *t*‐tests. Tumor growth curves (B left panel) were analyzed using repeated‐measures two‐way ANOVA followed by Bonferroni's post hoc test. Significance is denoted as: *p < 0.05, **p < 0.01, ***p < 0.001, ****p < 0.0001. Sample size (*n* = 5) represents biological replicates (individual mice/tumors) per group. All statistical analyses were performed using GraphPad Prism or R software.

We next evaluated the antitumor efficacy of rimegepant in immunocompetent murine models. In mice bearing EO771 tumors, rimegepant given on a defined schedule significantly suppressed tumor progression and reduced final tumor mass relative to vehicle‐treated controls (Figure 6A,B). This growth inhibition was accompanied by substantial remodeling of the tumor immune landscape, characterized by increased total leukocyte (CD45^+^) infiltration (Figure 6C).

Pharmacodynamic analysis confirmed on‐target mechanism engagement in vivo: IHC staining revealed a significant decrease in intra‐tumoral ARID3A protein levels and a concurrent increase in the density of CD8^+^GZMB^+^ cytotoxic T cells in rimegepant‐treated tumors (Figure S5C,D). The treatment was well‐tolerated, with no significant body weight loss (Figure S5E), abnormal serum biochemistries (e.g., bilirubin, urea, liver enzymes; Figure S5F), or histopathological toxicity in major organs (lung, spleen, liver, heart, kidney; Figure S5G).

We next used high‐dimensional flow cytometry of tumor‐infiltrating lymphocytes to profile rimegepant's effects, which revealed expanded activated (CD69^+^CD44^+^) and highly functional CD8^+^ T cells. This was evidenced by increased frequencies of IFN‐γ^+^, TNF‐α^+^, and GZMB^+^ cells and decreased PD‐1^+^TIM3^+^ cells (Figure 6D–I). To resolve these changes at single‐cell resolution, we performed scRNA‐seq. UMAP visualization showed marked reconfiguration of the T cell compartment in rimegepant‐treated tumors: expansion of effector‐like and tissue‐resident memory (TRM)‐like CD8^+^ T cell clusters, and contraction of exhausted, dysfunctional subsets (Figure 6J–N; Figure S6A–D). Transcriptomic analysis confirmed enrichment of cytotoxic gene programs (*Cd8, Gzmb, Ifng, Prf1*) and interferon‐responsive genes (*Isg15, Tnf*) in the CD8^+^ T cell lineage, while myeloid and stromal compartments had upregulated antigen‐processing and interferon‐stimulated gene (ISG) modules (Figure 6O, Figure S6E).

In summary, rimegepant elicited a well‐tolerated, mechanism‐driven antitumor response, characterized by suppressed tumor growth and comprehensive reprogramming of the TME toward an interferon‐rich, cytotoxic state.

### 2.7 Rimegepant Synergizes with PD‐1 Checkpoint Blockade

**FIGURE 7:**
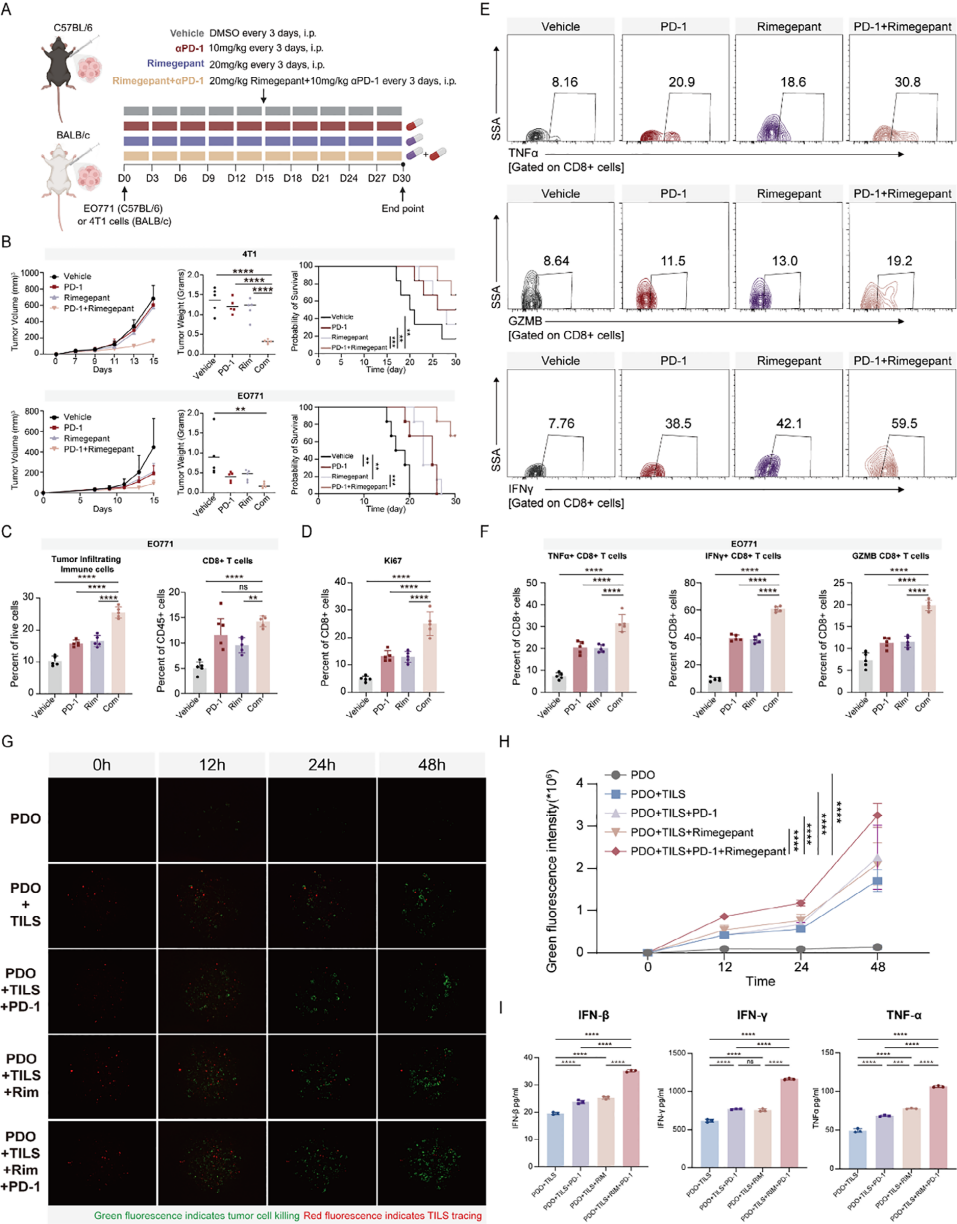
Rimegepant synergizes with anti–PD‐1 therapy in mouse models and patient‐derived organoids. (A) Schematic illustration of the in vivo combination treatment regimen. Mice bearing EO771 or 4T1 tumors were treated with vehicle control, anti‐PD‐1 antibody (αPD‐1, labeled as PD‐1 in figures), Rimegepant (Rim), or the combination of both, as indicated. Created with BioRender.com (Agreement No. UM2944GNI4). (B) Combination therapy maximizes tumor growth inhibition in vivo. Tumor growth kinetics (left), final tumor weights (middle), and Kaplan‐Meier survival analysis (right) in mice treated as indicated (*n* = 5 mice per group). Tumor growth curves were analyzed using repeated‐measures two‐way ANOVA with Bonferroni's post‐hoc test. Final tumor weights were analyzed using one‐way ANOVA. Survival differences were evaluated using the log‐rank test. (C, D) Flow cytometry analysis and quantification of (C) total tumor‐infiltrating immune cells (CD45^+^, left) and CD8^+^ T cells (right), and (D) proliferating CD8^+^ T cells assessed by Ki‐67 expression, in tumors from the indicated treatment groups (*n* = 5 tumors per group). Com indicates the combination therapy group (Rim + αPD‐1). (E, F) Combination therapy enhances CD8^+^ T cell effector function in vivo. (E) Representative flow cytometry contour plots and (F) quantification of TNF‐α^+^, IFN‐γ^+^, and granzyme B (GZMB)^+^ CD8^+^ T cells within the tumor microenvironment (*n* = 5 tumors per group). (G, H) Rimegepant and αPD‐1 synergistically promote tumor killing in vitro. (G) Time‐lapse fluorescence microscopy of patient‐derived tumor organoids (PDOs) co‐cultured with autologous tumor‐infiltrating lymphocytes (TILs) under the indicated conditions. Green fluorescence indicates tumor cell death; red fluorescence traces TILs. (H) Quantification of total green fluorescence intensity over time, reflecting cumulative tumor cell lysis (*n* = 3 biologically independent PDO cultures). Data were analyzed using two‐way ANOVA. (I) Enzyme‐linked immunosorbent assay (ELISA) measurement of IFN‐β, IFN‐γ, and TNF‐α secretion levels in the PDO‐TIL co‐culture supernatants from the indicated treatment groups (*n* = 3 biologically independent samples per group). Statistical Analysis: All quantitative data are presented as mean ± SEM. Statistical comparisons among multiple treatment groups were performed using one‐way ANOVA (for single time‐point assays) or two‐way ANOVA (for time‐course assays) followed by Bonferroni's post‐hoc test. In ANOVA analyses, statistical significance specifically denotes comparisons between the combination therapy group and the other single‐agent or vehicle groups, unless otherwise indicated by connecting lines. Survival analyses were conducted using the log‐rank test. Significance is denoted as: *p < 0.05, **p < 0.01, ***p < 0.001, ****p < 0.0001; ns, not significant. All statistical analyses were performed using GraphPad Prism software.

Given its capacity to reprogram the tumor immune microenvironment, we hypothesized that rimegepant could overcome primary resistance to ICB. In both EO771 and 4T1 syngeneic models, the combination of rimegepant with anti–PD‐1 therapy demonstrated markedly superior efficacy compared to either agent alone, resulting in significantly delayed tumor growth, reduced final tumor weights, and substantially prolonged host survival (Figure 7A,B).

Immune profiling of treated tumors revealed the mechanistic basis for this synergy. The combination drove the highest total leukocyte and CD8^+^ T cell infiltration (Figure 7C). Among CD8^+^ T cells, we observed enhanced proliferation (Ki‐67^+^) and a further increase in the frequency of polyfunctional effector cells producing IFN‐γ, TNF‐α, and GZMB (Figure 7D–F). Crucially, the combination also reduced co‐expression of the inhibitory receptors PD‐1 and TIM‐3, indicating reduced T cell exhaustion and dysfunction (Figure 7H).

To evaluate translational relevance, we employed patient‐derived tumor organoids (PDOs) co‐cultured with autologous T cells. Live‐cell imaging and quantitative killing assays demonstrated significantly enhanced tumor cell lysis in the rimegepant plus anti–PD‐1 group compared to monotherapies (Figure 7G,H). Cytokine profiling of co‐culture supernatants corroborated these findings, showing elevated levels of IFN‐γ, IFN‐β, and TNF‐α in the combination group, consistent with coordinated activation of the innate‐adaptive immune axis (Figure 7I).

Collectively, these results demonstrate that pharmacological targeting of the ARID3A program with rimegepant synergizes with PD‐1 blockade to amplify a potent and sustained CD8^+^ T cell response, offering a promising combination strategy to circumvent resistance to immune checkpoint inhibition.

## 3 Discussion

Our study identified a targetable immune‐evasion program in TNBC orchestrated by ARID3A. We showed that tumor‐intrinsic ARID3A contributes to the maintenance of an immune‐cold microenvironment by restraining nucleic‐acid sensing and downstream IFN‐I signaling, thereby limiting dendritic cell activation and antigen‐specific CD8^+^ T‐cell responses. In addition, structure‐guided mutational analyses provided genetic evidence supporting on‐target engagement of rimegepant with ARID3A, establishing a mechanistic basis for pharmacologic modulation of this pathway.

TNBC remains a clinically challenging subtype characterized by immune exclusion and limited responsiveness to ICB [1]. While ARID3A has been implicated in cancer progression in other contexts, its role in regulating tumor immunogenicity has been unclear. Our findings position ARID3A as a key determinant of immune exclusion in TNBC. *ARID3A* is frequently upregulated, inversely correlates with cytotoxic T‐cell infiltration, and associates with poor survival and primary resistance to ICB, suggesting its potential utility as a biomarker complementary to PD‐L1. Importantly, *Arid3a* deficiency does not affect intrinsic tumor cell fitness but instead enhances CD8^+^ T cell–mediated tumor control, consistent with a tumor‐intrinsic immunoregulatory role.

Mechanistically, we identified ARID3A as a suppressor of tumor cell–intrinsic innate immune activation, a role distinct from its reported function in promoting IFN‐I signaling in autoimmune contexts [30]. In TNBC, genetic ablation of *Arid3a* impairs DNA repair and increases cytosolic dsDNA, thereby activating the cGAS–STING axis. In parallel, transcriptional upregulation of the kinase IKBKE potentiates signaling downstream of the RIG‐I–MAVS RNA‐sensing pathway [31]. Together, these pathways converge to drive robust IFN‐I responses, accompanied by induction of interferon‐stimulated genes and T cell–recruiting chemokines such as *Cxcl9* and *Cxcl10*. We further observed upregulation of IFI200 family members (e.g., IFI209 and IFI205), known positive regulators of cGAS–STING, suggesting a feed‐forward circuit that may sustain innate immune activation even under limiting ligand conditions [32, 33]. Critically, this antitumor immunity is abolished by IFNAR1 blockade or CD8^+^ T cell depletion, establishing the IFN‐I–CD8^+^ T cell axis as essential for tumor control. These findings provide a mechanistic explanation for the immune‐cold phenotype of *ARID3A*‐high TNBC, in which suppression of nucleic‐acid sensing limits the initiation of adaptive immune responses.

Beyond tumor‐intrinsic effects, *Arid3a* deficiency reshapes the tumor immune landscape by enhancing the recruitment and activation of DC‐enriched populations, as evidenced by increased surface CD80/CD86 expression. This immune‐permissive microenvironment promotes more effective CD8^+^ T cell priming and effector function, consistent with the established role of tumor‐intrinsic IFN‐I in licensing dendritic cell–mediated antitumor immunity. Thus, ARID3A suppression coordinately enhances tumor immunogenicity and innate immune activation to drive a potent antitumor response.

An important unresolved question concerns the origin of cytosolic dsDNA accumulation following ARID3A loss. Our transcriptomic and biochemical data support an integrative model wherein the loss of ARID3A impairs DNA damage repair (DDR) programs, leading to unresolved double‐strand breaks and genomic instability (as evidenced by elevated γH2AX). This genomic stress likely promotes the formation of micronuclei—a well‐established and potent source of cytosolic DNA. In parallel, the loss of genome integrity and potential derepression of endogenous retroelements may provide additional nucleic acid ligands for cGAS and RIG‐I–MAVS sensing. Future studies will be required to quantitatively dissect the relative contributions of these sources (e.g., through detailed micronuclei quantification and retroelement transcriptomics) and to define the direct transcriptional targets of ARID3A that govern nucleic‐acid homeostasis.

More broadly, our results offer a unifying perspective on ARID3A's context‐dependent functions. Its established role in maintaining cellular identity and transcriptional homeostasis—during embryonic development and B‐cell lineage commitment [30]—aligns conceptually with our observations in TNBC. Here, ARID3A appears to enforce a low‐immunogenicity “self” state that can be co‐opted by cancer cells to evade immune detection. This framework redefines ARID3A not merely as an oncogene or tumor suppressor, but as a guardian of cellular identity with important consequences for tumor immunogenicity.

A key translational implication of our study is that this immune‐evasion program is pharmacologically tractable. Through screening of an FDA‐approved compound library, we identified rimegepant as an ARID3A‐targeting modulator that phenocopies *Arid3a* deficiency, activates IFN‐I signaling, remodels the tumor microenvironment, and synergizes with anti–PD‐1 therapy to eradicate otherwise resistant tumors. In murine models, rimegepant administered at 20 mg/kg intraperitoneally corresponds to a human equivalent dose of approximately 1.62 mg/kg (∼97 mg for a 60‐kg adult) based on body surface area normalization [34, 35, 36]. This estimate is comparable to the clinically approved 75 mg oral dose used for acute migraine treatment [37, 38, 39], supporting the translational feasibility of this approach, although dedicated oncology‐focused pharmacokinetic and pharmacodynamic studies will be required.

Convergent evidence—including computational docking, phenotypic mimicry of *Arid3a* deficiency, and pocket‐mutant rescue experiments—supports a model in which rimegepant exerts its immunostimulatory effects through direct engagement of ARID3A. Mutations of predicted pocket residues (Y326 or R266) markedly attenuate rimegepant‐induced IFN signaling and tumor‐cell responses, providing direct genetic evidence of on‐target activity. Although rimegepant is a known CGRP receptor antagonist, potential CGRP‐dependent effects in tumor or immune compartments warrant further investigation. Nonetheless, our data establish ARID3A as a principal mechanistic node underlying its anticancer activity.

Several important questions remain. Future studies should delineate how ARID3A regulates DNA repair fidelity, replication stress, and nucleic‐acid homeostasis, and determine whether specific immune subsets—such as cDC1 populations involved in cross‐presentation—are particularly dependent on this axis. It will also be important to evaluate rimegepant‐based combinations in biomarker‐selected, *ARID3A*‐high tumors and to define exposure–response relationships in oncology‐focused studies.

Importantly, the relevance of this pathway may extend beyond TNBC. Pan‐cancer analyses suggest that *ARID3A*‐high, immune‐cold tumors represent a recurrent phenotype across multiple malignancies, raising the possibility that pharmacologic targeting of the ARID3A–IFN axis could broaden the efficacy of ICB in otherwise nonresponsive cancers. Consistent with this, *ARID3A* expression is detectable in ER‐positive and HER2‐positive breast cancers, albeit within distinct immunologic contexts. Although speculative, ARID3A‐mediated suppression of innate immune sensing may cooperate with hormone‐driven immune exclusion in ER‐positive disease or attenuate immune‐dependent therapeutic responses in HER2‐positive tumors.

In summary, we identify ARID3A as a central tumor‐intrinsic regulator of immune exclusion and establish the ARID3A–IFN axis as a therapeutically actionable vulnerability. These findings provide a strong mechanistic rationale for combining ARID3A‐targeting strategies with immune checkpoint blockade to overcome resistance in in *ARID3A*‐high, immune‐cold tumors.

## 4 Materials and Methods

### 4.1 Cell Lines and Culture

The mouse breast cancer cell lines EO771, Py8119, and 4T1 were obtained from ATCC in 2024: EO771 (ATCC Cat# CRL‐3461, RRID: CVCL_GR23), Py8119 (ATCC Cat# CRL‐3278, RRID: CVCL_AQ09), and 4T1 (ATCC Cat# CRL‐2539, RRID: CVCL_0125). All cell lines were maintained in DMEM (Gibco) supplemented with 10% fetal bovine serum and 1% penicillin‐streptomycin at 37°C in a humidified incubator with 5% CO_2_. All cultures were routinely tested and confirmed to be mycoplasma‐free.

### 4.2 Generation of OVA‐Expressing Stable Cell Lines

Stable OVA‐expressing EO771 and Py8119 cells were established via lentiviral transduction. Briefly, 293T packaging cells were co‐transfected with the transfer plasmid pLenti‐CMV‐OVA‐ZsGreen and the packaging plasmids psPAX2 and pMD2.G using a standard calcium phosphate protocol. Lentiviral supernatants were collected 48 h post‐transfection, centrifuged at 1500 × g for 5 min to remove cell debris, and filtered through a 0.45 µm membrane. Target cells were then incubated with the viral supernatant in the presence of polybrene (8 µg/mL) for 48 h. ZsGreen‐positive cells were isolated by fluorescence‐activated cell sorting (FACS) on a BD FACSAria III sorter and expanded for subsequent experiments. To ensure a homogeneous population, this sorting procedure was repeated three times for each cell line.

### 4.3 Animals

Six‐week‐old female BALB/c nude, C57BL/6, and NSG mice were purchased from Shanghai Jihui Laboratory Animal Breeding Co., Ltd. All mice were housed in a specific pathogen‐free (SPF) environment under controlled conditions, with a temperature of 20–22°C and relative humidity of 60 ± 10%. Regular monitoring, including serological testing and microscopic examination, was performed to ensure animal health.

For the establishment of the orthotopic tumor model, 2 × 10^6^ EO771 cells were inoculated into the fourth inguinal mammary fat pad of female C57BL/6 mice and nude mice, respectively. Separately, 4 × 10^6^ 4T1 cells were inoculated into BALB/c and nude mice, respectively. Tumor volumes were measured using a vernier caliper and calculated with the formula: 0.52 × length × width^2^.

All animal experiments were conducted in strict accordance with protocols approved by the Research Ethics Committee of Fudan University Shanghai Cancer Center (FUSCC; Approval No. FUSCC‐IACUC‐2023275).

### 4.4 Tumor Sample Processing and Single‐Cell Suspension Preparation

Resected tumor tissues were washed twice with ice‐cold PBS containing 1% BSA (Sigma‐Aldrich) and minced into 2–4 mm^3^ fragments. The fragments were enzymatically dissociated using the Mouse Tumor Dissociation Kit (Miltenyi Biotech) according to the manufacturer's instructions. The resulting cell suspension was filtered through a 70 µm SmartStrainer (Miltenyi Biotech). Red blood cells were lysed using a 1× Red Blood Cell Lysis Solution (Miltenyi Biotech), and dead cells were removed with the Dead Cell Removal Kit (Miltenyi Biotech). The final live cell suspension was washed twice, resuspended in an appropriate buffer, and used for downstream single‐cell experiments.

### 4.5 Culture of TNBC Patient‐Derived Organoids (TNBC‐PDOs)

Approximately 1.5×10^6^ single cells per milliliter were washed with Advanced DMEM/F12 (Invitrogen) and embedded in 20 µL of Matrigel (BD Bioscience) in a 48‐well plate to generate TNBC patient‐derived organoids. The organoids were maintained in OneCULTar breast tumor culture medium (Org‐A1‐02), with the medium being refreshed every three to four days. Passaging was performed approximately every seven days. For subculturing, the organoids were dissociated by incubation in 200 µL of TrypLE Express (GIBCO) for 1–3 min, followed by mechanical pipetting. The resulting fragments were washed at least twice to remove residual Matrigel and then resuspended in fresh Matrigel for reseeding in culture plates. Throughout the culture period, optical images of the TNBC‐PDOs were captured to monitor morphological development.

### 4.6 Flow Cytometry and Gating Strategy

For cell surface staining, single‐cell suspensions were adjusted to a density of 1×10^7^ cells/mL and incubated with anti‐mouse CD16/32 (Fc block, BioLegend, Clone 93, Cat# 101302) for 20 min at 4°C to prevent non‐specific binding. Cells were then stained for 30 min at 4°C in the dark with fluorochrome‐conjugated antibodies against the following targets: CD45 (PE/Cyanine7, BioLegend, Clone 30‐F11, Cat# 103114), CD11c (APC/Fire 750, BioLegend, Clone N418, Cat# 117348), CD3ε (FITC, BioLegend, Clone 145‐2C11, Cat# 100306), CD4 (Brilliant Violet 510, BioLegend, Clone RM4‐5, Cat# 100449), CD8a (Brilliant Violet 421, BioLegend, Clone 53‐6.7, Cat# 100753), CD25 (APC, BioLegend, Clone PC61, Cat# 102012), CD80 (Brilliant Violet 650, BioLegend, Clone 16‐10A1, Cat# 104728), CD86 (Brilliant Violet 711, BioLegend, Clone GL‐1, Cat# 105034), CD44 (PE, BioLegend, Clone IM7, Cat# 103008), PD‐1 (FITC, BioLegend, Clone 29F.1A12, Cat# 135214), TIM‐3 (APC, BioLegend, Clone RMT3‐23, Cat# 119706), and CD69 (PE/Cyanine7, BioLegend, Clone H1.2F3, Cat# 104512), which were used in separate staining panels where applicable to avoid fluorophore overlap. Cell viability was assessed using the Fixable Viability Dye Red 780 (Elabscience, Cat# E‐CK‐A168; displayed as “LD R780” in all flow cytometry plots). In selected experiments, cell suspensions from EO771‐OVA models were incubated with PE‐labeled H‐2K^b^ OVA (SIINFEKL) tetramer (Yeasen Biotechnology, Cat# 13903) for 30 min at room temperature in the dark prior to surface antibody staining.

For intracellular cytokine staining (ICS), single‐cell suspensions were stimulated with PMA and ionomycin in the presence of protein transport inhibitors (e.g., Brefeldin A) prior to fixation and permeabilization using the Foxp3/Transcription Factor Staining Buffer Set (eBioscience). Cells were subsequently stained with antibodies against IFN‐γ (APC, BioLegend, Clone XMG1.2, Cat# 505810), Granzyme B (PerCP/Cyanine5.5, BioLegend, Clone GB11, Cat# 372212), TNF‐α (Brilliant Violet 510, BioLegend, Clone MP6‐XT22, Cat# 506339), Ki‐67 (Brilliant Violet 605, BioLegend, Clone 16A8, Cat# 652413), and FOXP3 (Alexa Fluor 700, eBioscience, Clone 150D, Cat# 126422).

Data acquisition was performed based on assay complexity: high‐dimensional multicolor analyses of tumor‐infiltrating immune cells were acquired on a BD LSRFortessa flow cytometer, whereas selected low‐parameter in vitro assays were acquired on a BD Accuri C6 flow cytometer. Fluorescence compensation was performed using single‐stained controls. Fluorochrome conjugates shown in representative flow cytometry plots reflect the actual acquisition channels used for each panel and may differ from those listed above due to panel‐specific optimization. All flow cytometry data were analyzed using FlowJo software (v10.8.1). Positive gating boundaries were established using fluorescence minus one (FMO) controls.

Representative gating strategies for tumor‐infiltrating immune cells are provided in Supplementary Figure S7. Briefly, debris was excluded based on FSC‐A versus SSC‐A, followed by doublet exclusion using FSC‐A versus FSC‐H. Viable cells were identified as LD R780‐negative populations, followed by the positive selection of CD45^+^ leukocytes for downstream analysis.

For T cell analysis, sequential gating was performed to identify lymphocytes, singlets, viable cells, and CD45^+^ leukocytes. For the analysis of surface activation (CD44^+^, CD69^+^) and exhaustion (PD‐1^+^TIM‐3^+^) markers (without in vitro stimulation), CD8^+^ T cells were identified within the CD3^+^ population (final gate: CD45^+^CD3^+^CD8^+^). For intracellular effector function (GZMB^+^, IFN‐γ^+^, TNF‐α^+^) and proliferation (Ki‐67^+^) analysis, where activation‐induced CD3 downregulation is well‐documented following PMA/ionomycin stimulation, CD8^+^ T cells were gated directly from the viable CD45^+^ leukocyte population (final gate: CD45^+^CD8^+^) to prevent the loss of activated effector T cells. In selected experiments, tetramer‐positive cells were quantified as a percentage of CD8^+^ T cells. For dendritic cell (DC) analysis, live CD45^+^CD11c^+^ cells were gated as DC‐enriched populations, and CD80/CD86 co‐expression was quantified to assess maturation.

### 4.7 T Cell Co‐Culture and Cytotoxicity Assays

For antigen‐specific T cell activation, 1×10^5^ EO771‐OVA or Py8119‐OVA cells were seeded per well in 96‐well plates and allowed to adhere for 12 h. CD8^+^ T cells were isolated from the spleens of OT‐I transgenic mice using the EasySep Mouse CD8^+^ T Cell Isolation Kit. Purified OT‐I T cells were then added to the tumor cells at an effector‐to‐target (E:T) ratio of 5:1 in complete medium supplemented with IL‐2 (10 ng/mL). Brefeldin A (1:1000 dilution) was added for the final 4 h of co‐culture to block cytokine secretion. Cells were subsequently harvested for flow cytometric analysis of T cell activation and effector molecules.

To evaluate OVA‐specific cytotoxicity, splenocytes derived from OT‐I TCR transgenic mice were first cultured in complete RPMI 1640 medium (containing 10% fetal bovine serum, 1% penicillin‐streptomycin, 0.05 mM 2‐mercaptoethanol, and 10 ng/mL IL‐2) and stimulated with OVA peptide (10 nM). After 48 h, the cells were washed to remove the OVA peptide and rested in complete RPMI 1640 medium for an additional 72 h. These activated OT‐I CTLs were then co‐cultured with OVA‐expressing target cells for 12 h. Target cell viability was subsequently quantified using a fluorescence‐based readout.

### 4.8 Immunoblotting

Total protein was extracted from cells or snap‐frozen tissues using RIPA lysis buffer (Thermo Fisher Scientific) supplemented with protease and phosphatase inhibitors; lysates were centrifuged at 15,000 × g for 15 min at 4°C, and protein concentration was determined using a BCA assay kit (Thermo Fisher Scientific). Equal amounts of protein were separated by SDS‐PAGE and transferred to PVDF membranes (Millipore), and after blocking with 5% non‐fat milk, the membranes were incubated overnight at 4°C with primary antibodies, including anti‐MAVS (1:1000, #24930 (D5A9E) Rabbit mAb, RRID:AB_2798889, Cell Signaling Technology), anti‐RIG‐I (1:800, 700366, RRID:AB_2532317, Thermo Fisher Scientific), anti‐IRF3 (1:500, 550428, RRID:AB_393677, BD Biosciences), anti‐phospho‐IRF3 (Ser396, 1:1000, #29047, RRID:AB_2773013, Cell Signaling Technology), anti‐TBK1 (1:800, #38066, RRID:AB_2827657, Cell Signaling Technology), anti‐phospho‐TBK1 (Ser172, 1:1000, #5483, RRID:AB_10693472, Cell Signaling Technology), anti‐STING (1:500, #50494, RRID:AB_2799375, Cell Signaling Technology), anti‐cGAS (1:1000, #15102, RRID:AB_2732795, Cell Signaling Technology), anti‐ARID3A (1:800, 14068‐1‐AP, RRID:AB_2060390, Proteintech), anti‐TUBULIN (1:5000, #3873, RRID:AB_1904178, Cell Signaling Technology), anti‐ACTIN (1:5000, #8457, RRID:AB_10950489, Cell Signaling Technology), anti‐dsDNA (1:500, ab27156, RRID:AB_470907, Abcam), anti‐dsRNA (1:800, #76651, RRID:AB_2936194, Cell Signaling Technology), anti‐CD8 (1:1000, #98941, RRID:AB_2756376, Cell Signaling Technology), anti‐GZMB (1:800, #4275, RRID:AB_2114432, Cell Signaling Technology), anti‐CD45 (1:500, #70257, RRID:AB_2799780, Cell Signaling Technology), and anti‐γ‐H2AX (Ser139, 1:1000, #9718, RRID:AB_2118009, Cell Signaling Technology). The membranes were then incubated with HRP‐conjugated secondary antibody (Proteintech), and protein expression was visualized and quantified using the Amersham Imager 600 (Cytiva).

### 4.9 Structure‐Guided Mutagenesis and Rescue Assays

Residue‐level free energy decomposition of the ARID3A–rimegepant complex identified R266 and Y326 as key candidate residues at the binding interface. Site‐directed mutagenesis was used to generate single (ARID3A‐R266A and ARID3A‐Y326A) and double (ARID3A‐R266A/Y326A) mutant constructs. An empty vector (Vector), wild‐type (WT) ARID3A, and the mutant constructs were then re‐expressed in ARID3A‐deficient cells. Comparable baseline expression of the WT and mutant ARID3A proteins was verified by immunoblotting prior to downstream evaluations of rimegepant‐induced protein degradation, IFN‐β secretion, and cell viability.

### 4.10 Biochemical Analysis

In mouse model, the plasma biochemical indicators, including alanine aminotransferase (ALT), aspartate aminotransferase (AST), uric acid (UA), Creatinine (CREA), and lactate dehydrogenase 1 (LDH1), were analyzed by a Cobas C8000 Automatic Biochemical Analysis System (Roche, Basel, Switzerland).

### 4.11 Hematoxylin–Eosin (HE) Staining

The tumor samples fixed in 10% formalin solution were embedded in paraffin, sectioned at a thickness of 4 µm, and then stained with HE. The pathological changes of each tissue sample were observed under a microscope (OLYMPUS BX61, Tokyo, Japan).

### 4.12 Immunofluorescence

Cultured cells were fixed with 4% formaldehyde for 10−15 min and then blocked with 3% BSA and 0.1% Triton X‐100 in PBS for 20 min at room temperature. Then immunostaining was performed. Nuclei were counterstained with DAPI. Images were taken with an Olympus FV1000 confocal microscope (Olympus, Japan) or Leica Inverted Confocal SP8 (Leica, Germany).

### 4.13 Immunohistochemistry

Tumor xenografts or surgical specimen tissue slides were deparaffinized, rehydrated through an alcohol series, followed by antigen retrieval with sodium citrate buffer. Tumor sections were blocked with 5% normal goat serum (Vector) with 0.1% Triton X‐100 and 3% H2O2 in PBS for 60 min at room temperature and then incubated with antibody (1:100, this study) overnight. Expression levels of those antigens were then detected by HRP‐conjugated DAB.

### 4.14 Single‐Cell RNA Sequencing and Data Analysis

Following the protocols outlined in the Chromium Next GEM Single Cell 3ʹ Reagent Kits v3 (10x Genomics, Pleasanton, CA, USA), we constructed cDNA libraries, which were subsequently pooled and sequenced on a NovaSeq 6000 platform (Illumina, San Diego, CA, USA).

FASTQ sequencing output files were demultiplexed and aligned to the mm10 genome using Cell Ranger (version 6.0.1). Downstream analysis of raw gene expression matrices was conducted using the Seurat R package (version 4.3.0). Cells were excluded if they met any of the following criteria: fewer than 200 unique genes expressed, more than 8,000 unique genes expressed, mitochondrial gene expression exceeding 10%, or hemoglobin gene expression exceeding 0.1%. Doublets were filtered out using the DoubletFinder R package (version 2.0.3). The remaining dataset comprised 46,603 high‐quality cells. Normalization of gene expression matrices was performed as previously described. Highly variable genes were selected using the FindVariableGenes function. Sample integration was achieved using IntegrateData, and the integrated dataset was subsequently scaled using ScaleData. To mitigate cell cycle effects, cell cycle scoring and regression were conducted following the methodology outlined in Seurat's online documentation (https://satijalab.org/seurat/articles/cell_cycle_vignette. html). Principal component analysis (PCA) was performed, and the top 30 principal components were selected for dimensionality reduction via uniform manifold approximation and projection (UMAP). Cell clustering was conducted using the FindClusters function in Seurat, and clusters were annotated based on the expression of canonical marker genes. For downstream analysis, cells from the two biological replicates in each group were integrated and analyzed as a pooled dataset per group using the Seurat “IntegrateData” function.

### 4.15 Bioinformatic Screening for Tumor‐Intrinsic Immune Regulators

A four‐tiered bioinformatic screening strategy was employed to identify potential tumor‐intrinsic regulators of immune evasion, adapted from a prior study [23]. The analysis integrated complementary immune‐related features as follows, using specified public databases. For all correlation analyses, genes were selected based on a correlation coefficient cutoff of |*r*|≥ 0.3 and a false discovery rate (*FDR*) < 0.05.

- Immune‐Privilege Signature Correlation: Genes positively correlated with established immune‐privilege signatures were identified using transcriptomic data from The Human Protein Atlas (HPA) database, aiming to capture factors that may establish a local tolerogenic niche.
- Negative Correlation with PD‐L1: Genes whose expression was negatively correlated with PD‐L1 (CD274) mRNA levels across cancer cell lines were derived from the Cancer Cell Line Encyclopedia (CCLE), to pinpoint potential checkpoint‐independent resistance mechanisms.
- Negative Association with IFN‐I Signaling: Genes exhibiting a negative association with IFN‐I signaling activity were analyzed using the TCGA pan‐cancer cohort, targeting putative suppressors of this innate immune initiation pathway.
- Negative association with IFN‐γ signaling: Similarly, genes negatively associated with IFN‐γ signaling were identified using the TCGA dataset, to find factors that may dampen the T cell effector phase.

### 4.16 Statistical Analysis

Data are presented as mean ± standard error of the mean (SEM) unless otherwise specified. No data were excluded from the analyses unless predefined quality‐control criteria were applied, as described for the single‐cell RNA sequencing data and in the corresponding figure legends. The sample size (*n*) represents biologically independent experiments, mice, tumors, patient samples, or PDO cultures, as indicated for each analysis.

For parametric tests, normal distribution and similar variances between groups were assumed based on the nature of the experimental data. Comparisons between two groups were performed using two‐tailed paired or unpaired Student's *t*‐tests, as appropriate. Comparisons involving three or more groups were analyzed using one‐way or two‐way ANOVA followed by Bonferroni's or Tukey's *post hoc* tests, as indicated. Tumor growth curves were analyzed using repeated‐measures two‐way ANOVA followed by Bonferroni's post hoc test. Survival analyses were performed using the Kaplan–Meier method with log‐rank testing, and correlation analyses were conducted using Pearson's correlation.

Visualization‐only single‐cell RNA‐seq panels were not subjected to formal hypothesis testing. A *p*‐value < 0.05 was considered statistically significant. Statistical analyses were performed using GraphPad Prism (version 10.4.0) or R (version 4.4.0). For visual clarity and consistency in the figures, mouse gene symbols used as labels (e.g., sg‐ARID3A) are presented in uppercase, although they refer to the corresponding mouse genes (e.g., *Arid3a*) as defined by MGI nomenclature guidelines.

## Data and Materials Availability

- All original sequencing data generated in this study will be deposited in the NCBI Gene Expression Omnibus (GEO) database and made publicly available prior to publication. The GEO accession number will be provided in the Key Resources Table of the final accepted manuscript. Until public release, raw data are available from the corresponding author upon reasonable request.
- Publicly available datasets analyzed in this study were obtained from The Cancer Genome Atlas (TCGA) GDC Data Portal (https://portal.gdc.cancer.gov/), the NCBI GEO repository (http://www.ncbi.nlm.nih.gov/geo), the Human Protein Atlas (https://www.proteinatlas.org/), and the Cancer Cell Line Encyclopedia (https://sites.broadinstitute.org/ccle/).
- All unique reagents generated in this study are available from the corresponding author upon reasonable request, subject to a material transfer agreement.
- Additional information required to reanalyze the data reported in this paper is available from the corresponding author upon reasonable request.
- This study does not report original custom code.

## Author Contributions

Conceptualization: Teng Zhou; Yifei Zhu; Jian Zhang; Methodology: Teng Zhou; Yifei Zhu; Jinlu Han; Cheng Zeng; Huiying Huang; Mingxi Lin; Yizi Jin; Qin Guo; Yuxin Yan; Xinhui Mao; Yifei Zhou; Doudou Li; Jian Zhang; Investigation: Teng Zhou; Yifei Zhu; Jinlu Han; Cheng Zeng; Jian Zhang; Visualization: Teng Zhou; Yifei Zhu; Jinlu Han; Cheng Zeng; Jian Zhang; Funding acquisition: Jian Zhang; Project administration: Jian Zhang; Supervision: Jinlu Han; Huiying Huang; Mingxi Lin; Yizi Jin; Jian Zhang; Writing – Original draft: Teng Zhou; Yifei Zhu; Writing – Review & editing: Teng Zhou; Yifei Zhu; Cheng Zeng; Jinlu Han; Huiying Huang; Doudou Li; Jian Zhang; Resources: Teng Zhou; Yifei Zhu; Jinlu Han; Huiying Huang; Mingxi Lin; Yizi Jin; Cheng Zeng; Qin Guo; Yuxin Yan; Xinhui Mao; Yifei Zhou; Doudou Li; Jian Zhang.

## Funding

The author(s) declare that financial support was received for the research, authorship, and/or publication of this article. This study was supported by the National Natural Science Foundation of China (Grant Number. 82373359); Shanghai Science and Technology Innovation Action Plan (Grant Number. 24SF1901700); CSCO Cancer Research Fund (grant nos. Y‐HR2020MS‐0298, Y‐pierrefabre202102‐0066); Chinese Young Breast Experts Research project (Grant Number. CYBER‐2021‐001); Beijing Science and Technology Innovation Medical Development Foundation Key Project (Grant Number. KC2022‐ZZ‐0091‐6).

## Ethics Statement

This study involving human participants was reviewed and approved by the Research Ethical Committee of Fudan University Shanghai Cancer Center (Approval No. 1612167‐18). Written informed consent was obtained from all participants. All animal experiments were conducted in accordance with protocols approved by the institutional Animal Care and Use Committee of Fudan University Shanghai Cancer Center (Approval No. FUSCC‐IACUC‐2023275).

## Consent

All authors have read and approved the final version of the manuscript and agree to its publication. Informed consent was obtained from all participants involved in the study.

## Conflicts of Interest

The authors declare no conflicts of interest.

## Supporting information




**Supporting File 1**: advs75399‐sup‐0001‐SuppMat.docx.

**Supporting File 2**: advs75399‐sup‐0002‐DataFile.pdf.

**Supporting File 3**: advs75399‐sup‐0003‐Data.zip.

## Data Availability

The data that support the findings of this study are available from the corresponding author upon reasonable request.
