# Supplementary material for: Pharmacologic Modulation of ARID3A with Rimegepant Reactivates Type I Interferon Signaling and Sensitizes Triple‐Negative Breast Cancer to PD‐1 Blockade
Source: Adv Sci (Weinh). 2026 Jun 2;13(40):e21541. doi: 10.1002/advs.202521541 (PMC13335518; doi:10.1002/advs.202521541)
Supplement: Supplementary file 1 — Supporting File 1: advs75399‐sup‐0001‐SuppMat.docx. [file ADVS-13-e21541-s001.docx]

**Supplemental Information Summary**


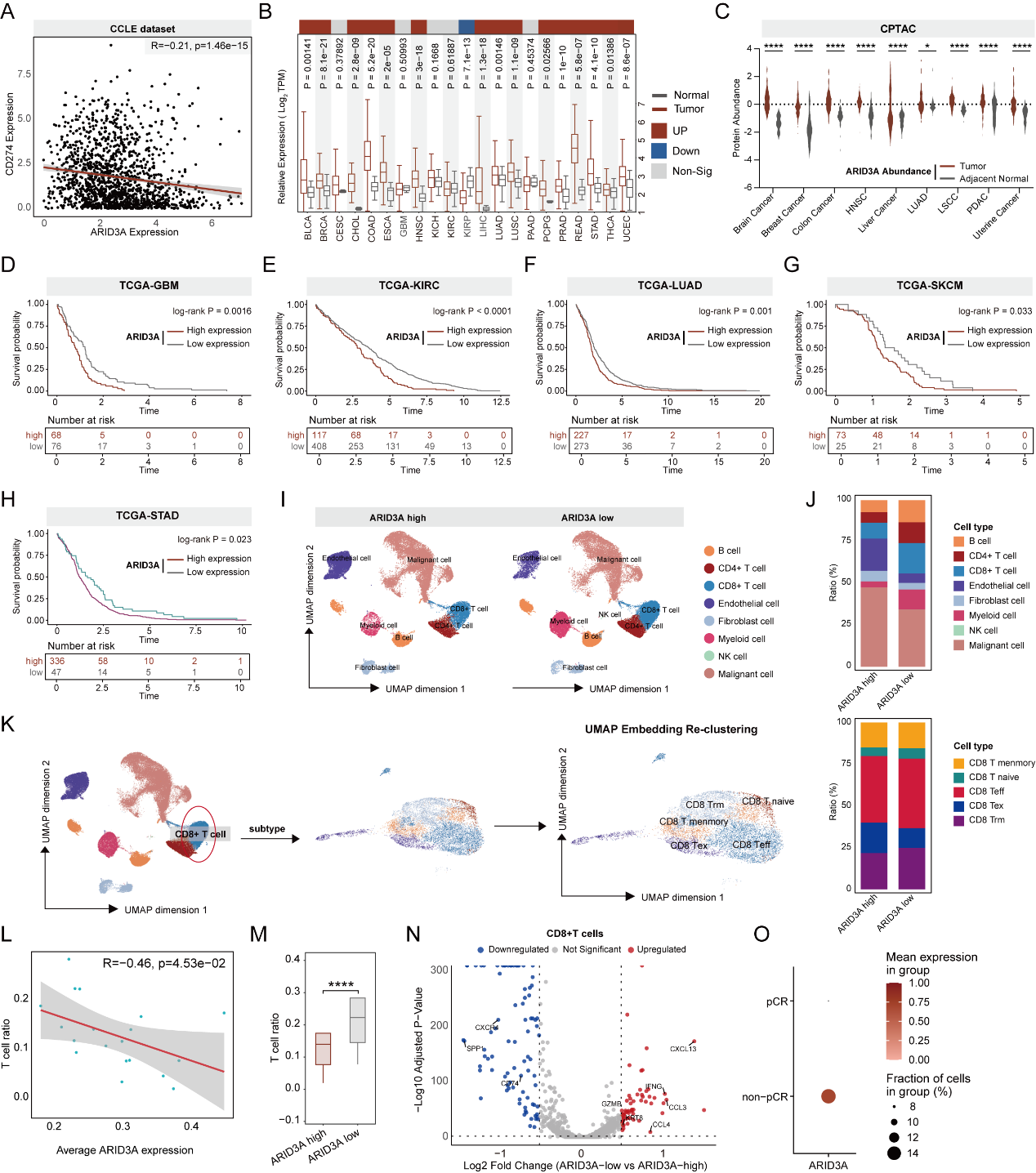


**Fig S1 | Pan-cancer analysis establishes ARID3A as a negative regulator of anti-tumor immunity.**

(A) *ARID3A* mRNA expression inversely correlates with *CD274* (PD-L1) expression across cancer cell lines in the Cancer Cell Line Encyclopedia (CCLE) dataset, as determined by Pearson correlation analysis (R = −0.21, P = 1.46 × 10⁻¹⁵).

(B, C) Dysregulation of ARID3A expression across human cancers. (B) *ARID3A* mRNA expression in tumor versus normal tissues across TCGA cohorts. (C) ARID3A protein abundance in tumor versus normal tissues across CPTAC cohorts. Statistical significance was determined by two-tailed unpaired Student’s t-tests.

(D–H) Kaplan-Meier analyses showing that high *ARID3A* expression is associated with poor overall survival in multiple TCGA cancer cohorts, including glioblastoma multiforme (GBM, D), kidney renal clear cell carcinoma (KIRC, E), lung adenocarcinoma (LUAD, F), skin cutaneous melanoma (SKCM, G), and stomach adenocarcinoma (STAD, H). P values were calculated using the log-rank test.

(I, J) Tumors with high *ARID3A* expression exhibit an immune-cold tumor microenvironment. (I) Uniform Manifold Approximation and Projection (UMAP) visualization of major tumor-infiltrating cell types. (J) Relative proportions of immune cell populations in *ARID3A*-high (n = 9) and *ARID3A*-low (n = 10) tumors, as determined by single-cell RNA sequencing (scRNA-seq).

(K) High *ARID3A* tumors harbor reduced cytotoxic CD8⁺ T cell subsets. UMAP visualization of CD8⁺ T cell subpopulations (left and middle) and corresponding relative proportions (right) in *ARID3A*-high versus *ARID3A*-low tumors.

(L, M) *ARID3A* expression negatively correlates with T cell infiltration. (L) Scatter plot showing the inverse correlation between *ARID3A* expression and T cell fraction in the GSE205506 cohort, as assessed by Pearson correlation analysis (R = −0.46, P = 4.53 × 10⁻²). (M) Quantification of CD8⁺ T cell proportions in *ARID3A*-high and *ARID3A*-low tumors. Statistical significance was determined by a two-tailed unpaired Student’s t-test.

(N) Volcano plot detailing transcriptomic profiling of CD8⁺ T cells from *ARID3A*-high versus *ARID3A*-low tumors, revealing upregulation of effector-associated genes and downregulation of exhaustion-associated markers in the *ARID3A*-low group.

(O) Dot plot showing *ARID3A* expression is lower in pathological complete responders (pCR, n = 15) compared with non-pCR patients (n = 4) following neoadjuvant anti-PD-1 therapy in colorectal cancer (CRC). Statistical significance was determined by a two-tailed unpaired Student’s t-test.

Statistical Analysis: Quantitative data are presented as mean ± SEM unless otherwise indicated. Box plots display the median, quartiles, and range. Comparisons between two independent groups were performed using two-tailed unpaired Student’s t-tests. Correlation analyses were conducted using Pearson correlation. Survival analyses were performed using the log-rank test. Significance is denoted as: *P < 0.05, **P < 0.01, ***P < 0.001, ****P < 0.0001. All statistical analyses were performed using GraphPad Prism or R software. For visual clarity in figures, gene symbols used as labels may be presented in regular uppercase, while they refer to the corresponding human genes (e.g., *ARID3A*) according to standard HGNC guidelines.


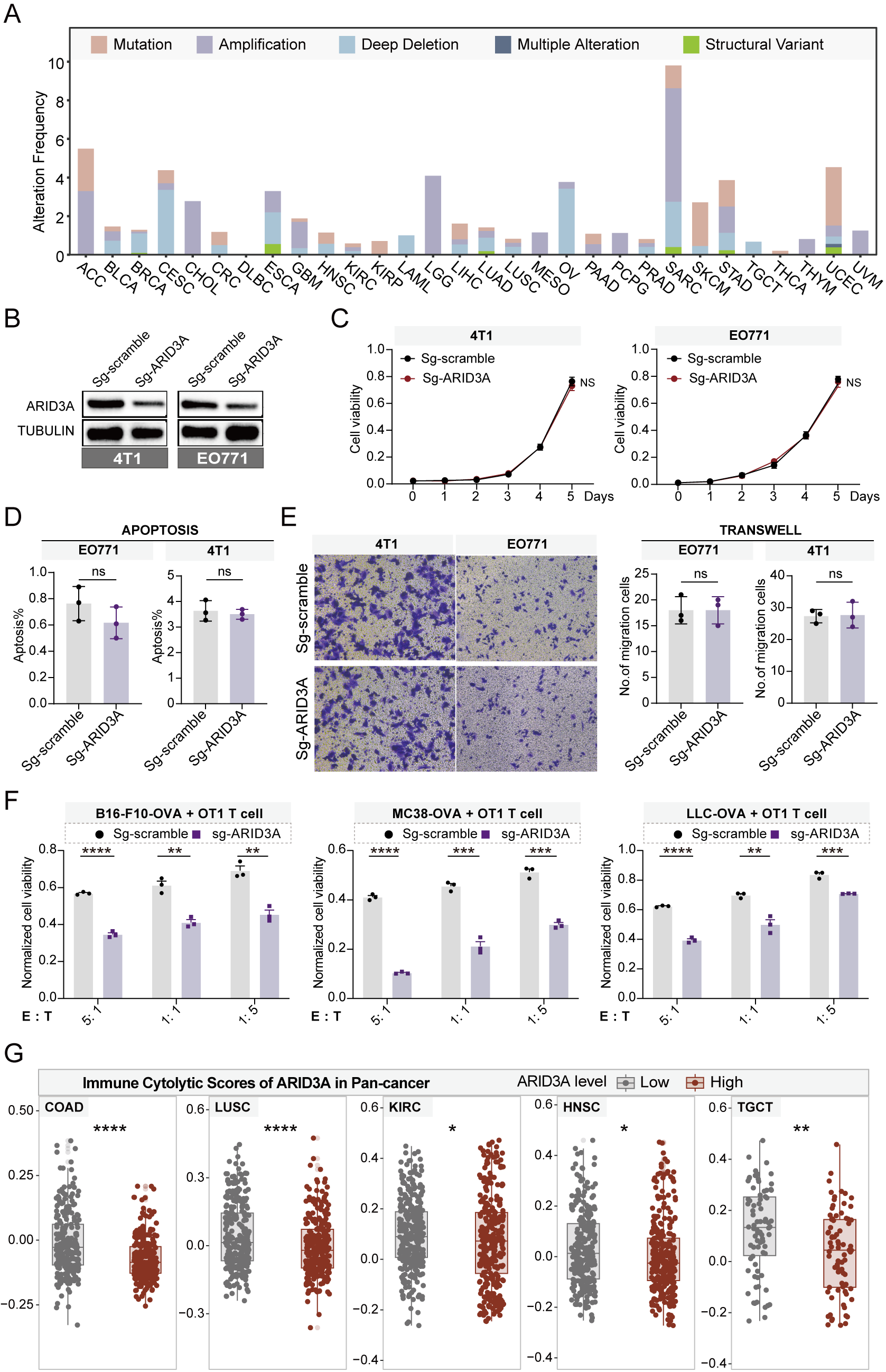


**Fig S2 | ARID3A alterations and their functional impact on tumor cell–intrinsic properties and T cell–mediated killing.**

(A) Frequency and spectrum of *ARID3A* genetic alterations across diverse cancer types derived from The Cancer Genome Atlas (TCGA) pan-cancer cohorts.

(B) Immunoblot validation of ARID3A knockout efficacy in EO771 and 4T1 cells stably expressing sg-*Arid3a* or sg-scramble constructs. Tubulin served as the loading control.

(C–E) *Arid3a* deficiency does not alter intrinsic tumor cell properties. (C) Cell proliferation, (D) apoptosis, and (E) transwell migration of sg-scramble- and sg-*Arid3a*-expressing EO771 and 4T1 cells. Representative images of the migration assay are shown. Data are presented as mean ± SEM (n = 3 biologically independent experiments). Statistical significance was determined by two-way ANOVA for proliferation assays and two-tailed unpaired Student’s t-tests for apoptosis and migration analyses.

(F) *Arid3a* deficiency enhances T cell-mediated cytotoxicity against multiple tumor cell lines. Viability of OVA-expressing tumor cells (B16-F10-OVA, MC38-OVA, and LLC-OVA) following co-culture with OT-I CD8⁺ T cells at the indicated effector-to-target (E:T) ratios (n = 3 biologically independent experiments). Statistical significance was determined by two-way ANOVA followed by Bonferroni’s post-hoc test.

(G) Tumors with low *ARID3A* expression exhibit significantly higher immune cytolytic scores across multiple TCGA cancer cohorts (COAD, LUSC, KIRC, HNSC, and TGCT). Statistical significance was determined by two-tailed unpaired Student’s t-tests.

Statistical Analysis: Quantitative data are presented as mean ± SEM. Statistical comparisons were performed using GraphPad Prism software. Comparisons between two independent groups were analyzed via two-tailed unpaired Student’s t-tests. Comparisons involving repeated measurements or multiple conditions (E:T ratios, time courses) were performed using two-way ANOVA with Bonferroni’s post-hoc test. Significance is denoted as: *P < 0.05, **P < 0.01, ***P < 0.001, ****P < 0.0001; ns, not significant. For visual clarity in figures, mouse gene symbols used as labels (e.g., Sg-ARID3A) are presented in uppercase, while they refer to the corresponding mouse genes (e.g., *Arid3a*) as defined by MGI guidelines.


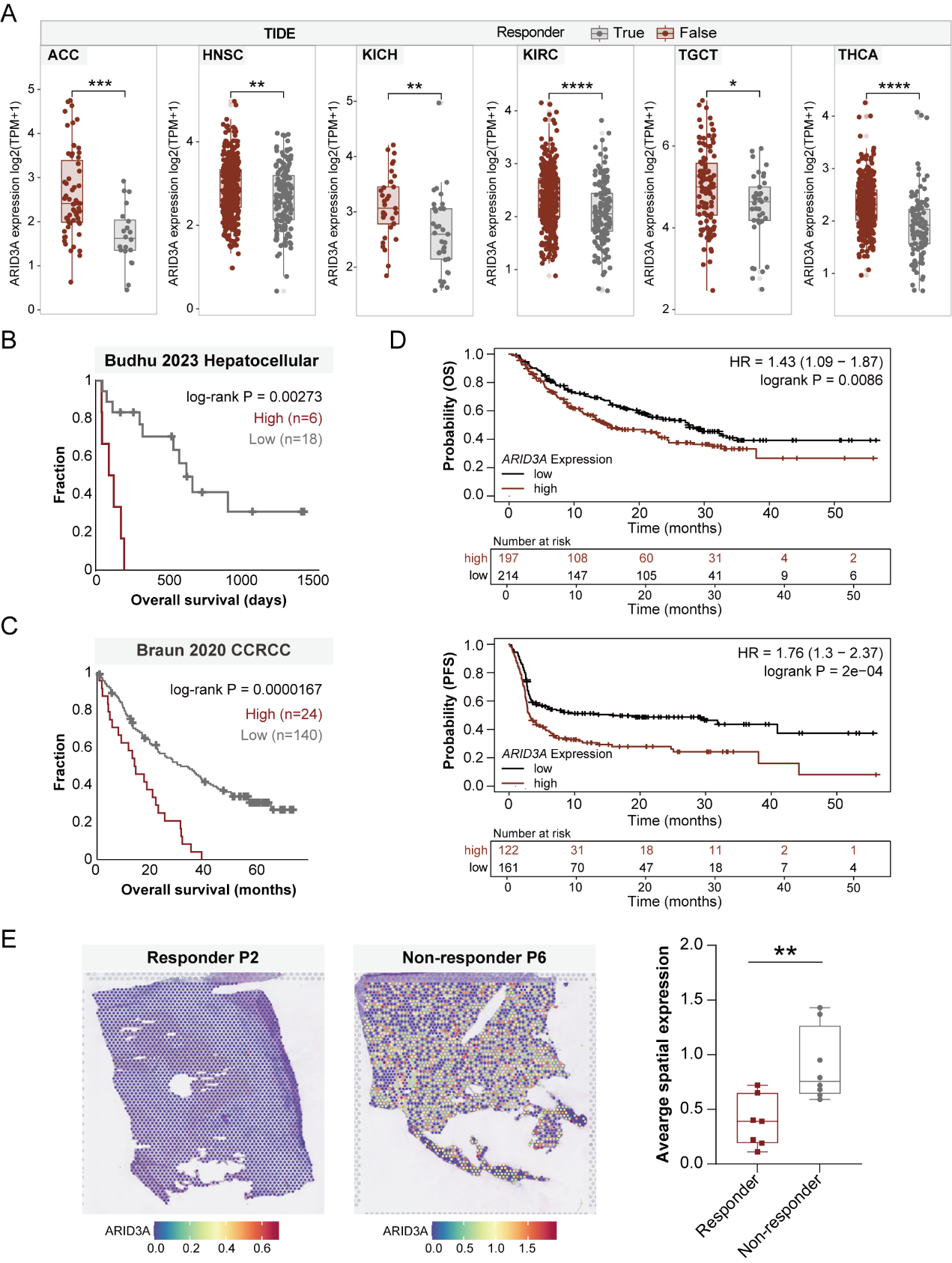


**Fig S3 | ARID3A expression predicts resistance to immune checkpoint blockade and poor clinical outcomes.**

(A) Tumor Immune Dysfunction and Exclusion (TIDE) analysis demonstrating lower *ARID3A* mRNA expression in immune checkpoint blockade (ICB) responders compared with non-responders across six cancer cohorts (ACC, HNSC, KICH, KIRC, TGCT, and THCA).

(B, C) Kaplan-Meier overall survival (OS) analyses of independent patient cohorts based on *ARID3A* expression levels. High *ARID3A* expression correlates with significantly worse OS in hepatocellular carcinoma (B, Budhu cohort) and clear cell renal cell carcinoma (C, Braun cohort). P values were calculated using the log-rank test.

(D) Cox proportional hazards regression analysis indicating that high *ARID3A* expression is a risk factor for both overall survival (OS; HR = 1.43) and progression-free survival (PFS; HR = 1.76) in ICB-treated patients.

(E) Spatial transcriptomics analysis comparing *ARID3A* expression distribution within the tumor microenvironment of an ICB responder (P2) and a non-responder (P6). The right panel shows the quantification of average spatial expression levels. Data in (E) are presented as a box-and-whisker plot (center line, median; box limits, upper and lower quartiles; whiskers, minimum to maximum values) with individual data points representing distinct spatial regions.

Statistical Analysis: Survival distributions (B, C) were compared using the log-rank test. Hazard ratios (D) were estimated using Cox proportional hazards regression models. Comparisons of continuous variables between two independent groups (A, E) were performed using two-tailed unpaired Student’s t-tests. Significance is denoted as: *P < 0.05, **P < 0.01, ***P < 0.001, ****P < 0.0001. All statistical analyses were performed using GraphPad Prism or R software.


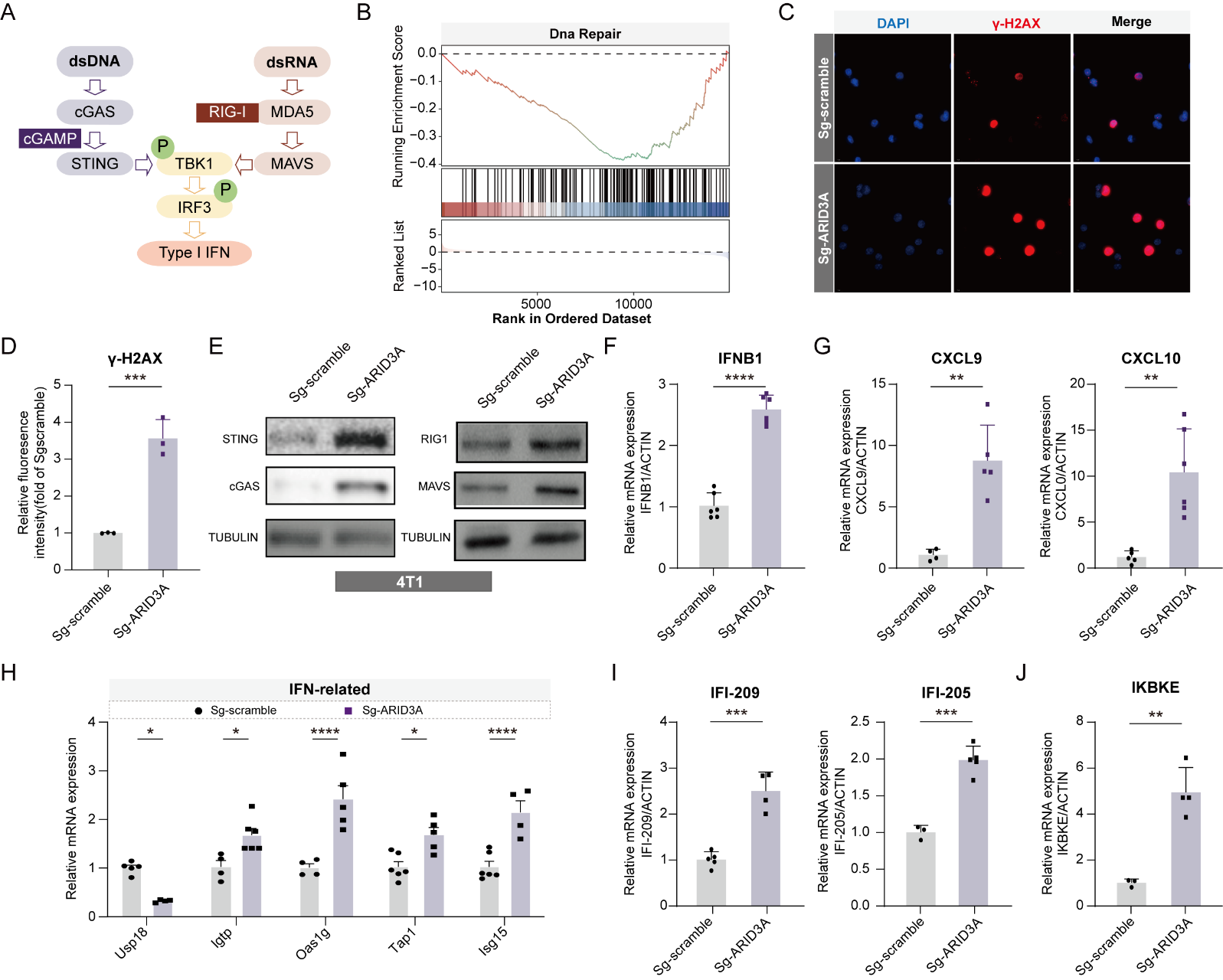


**Fig S4 | ARID3A suppression triggers DNA damage and innate immune activation.**

(A) Schematic representation of the cytosolic nucleic acid sensing pathways (cGAS-STING and RIG-I-MAVS) culminating in IRF3 phosphorylation and Type I Interferon (IFN) production. (B) GSEA plot showing the negative enrichment of the "DNA Repair" gene signature in *Arid3a*-deficient EO771 cells.

(C, D) *Arid3a* deficiency induces genomic instability. (C) Representative immunofluorescence images of γ-H2AX (red) and DAPI (blue) in EO771 cells. (D) Quantification of relative γ-H2AX fluorescence intensity (n = 3 independent biological replicates).

(E) Immunoblot analysis of RIG-I, MAVS, STING, and cGAS expression in sg-scramble- or sg-*Arid3a*-expressing 4T1 cells. Tubulin was used as the loading control.

(F–J) Transcriptional profiling of innate immune activation. qPCR analysis of (F) *Ifnb1*, (G) *Cxcl9* and *Cxcl10*, (H) IFN-related genes (*Usp18, Igtp, Oas1g, Tap1, and Isg15*), (I) *Ifi209* and *Ifi205*, and (J) *Ikbke* in EO771 cells (n = 5–6 independent biological replicates as indicated by individual data points). Data are normalized to *Actb*.

Statistical Analysis: Data are presented as mean ± SEM of at least three independent biological replicates (n values for specific panels are indicated by individual data points). Statistical significance between two groups was determined by a two-tailed unpaired Student’s t-test, or by two-way ANOVA with Bonferroni’s post-hoc test for multiple comparisons, using GraphPad Prism 9.0. Significance is denoted as: *P < 0.05, **P < 0.01, ***P < 0.001, ****P < 0.0001; ns, not significant.


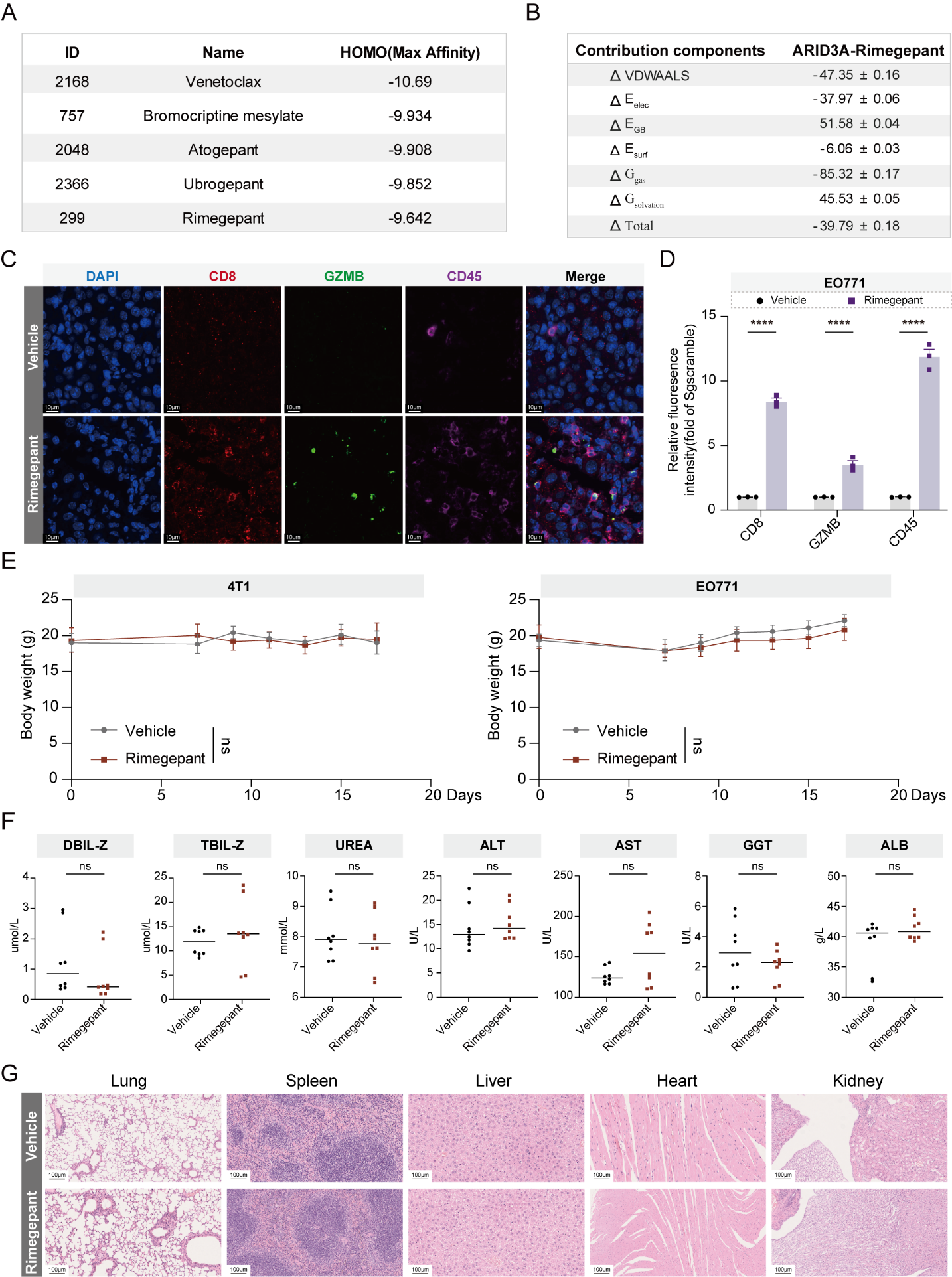


**Fig S5 | Rimegepant is a safe and efficacious ARID3A-targeting compound in vivo.**

(A) Top candidate compounds predicted to interact with ARID3A through computational virtual screening, ranked by their docking affinity scores (indicated as Max Affinity). (B) Molecular dynamics (MD) simulation data partitioning the binding free energy into specific components (van der Waals, electrostatic, etc.) for the ARID3A-Rimegepant complex. Data are expressed as mean ± SD from multiple simulation snapshots.

(C, D) Rimegepant promotes T cell infiltration and activation in vivo. (C) Representative immunofluorescence images of EO771 tumor sections. Tissues were stained for CD8 (red), GZMB (green), CD45 (magenta/purple), and DAPI (blue). Scale bars = 10 μm. (D) Quantitative analysis of fluorescence intensity for CD8, GZMB, and CD45 in EO771 tumors (n = 3 mice per group). Data represent relative fold change compared to the Sg-scramble control.

(E) Long-term safety assessment via body weight monitoring. Body weight curves of 4T1 and EO771 tumor-bearing mice treated with vehicle or Rimegepant (n = 5 mice per group). No significant differences were observed between groups (P > 0.05).

(F, G) Systematic toxicity evaluation. (F) Serum biochemical markers, including DBIL-Z, TBIL-Z, UREA, ALT, AST, GGT, and ALB, measured at the endpoint (n = 8 mice per group). Each dot represents an individual biological replicate. (G) Representative H&E staining of major organs (lung, spleen, liver, heart, and kidney) following treatment. Scale bars = 100 μm.

Statistical Analysis All quantitative data are presented as mean ± SEM. Sample size (n) represents biological replicates as indicated. Data normality was assessed via Shapiro-Wilk test. Comparisons between two groups were performed using two-tailed unpaired Student’s t-test. For longitudinal body weight, two-way repeated-measures ANOVA with Bonferroni’s post-hoc test was applied. Single-cell RNA-seq data were processed and analyzed using the Seurat (v4.0) pipeline in R. Statistical analyses and visualizations were performed using GraphPad Prism 9.0. Significance: *P < 0.05, **P < 0.01, ***P < 0.001, ****P < 0.0001.


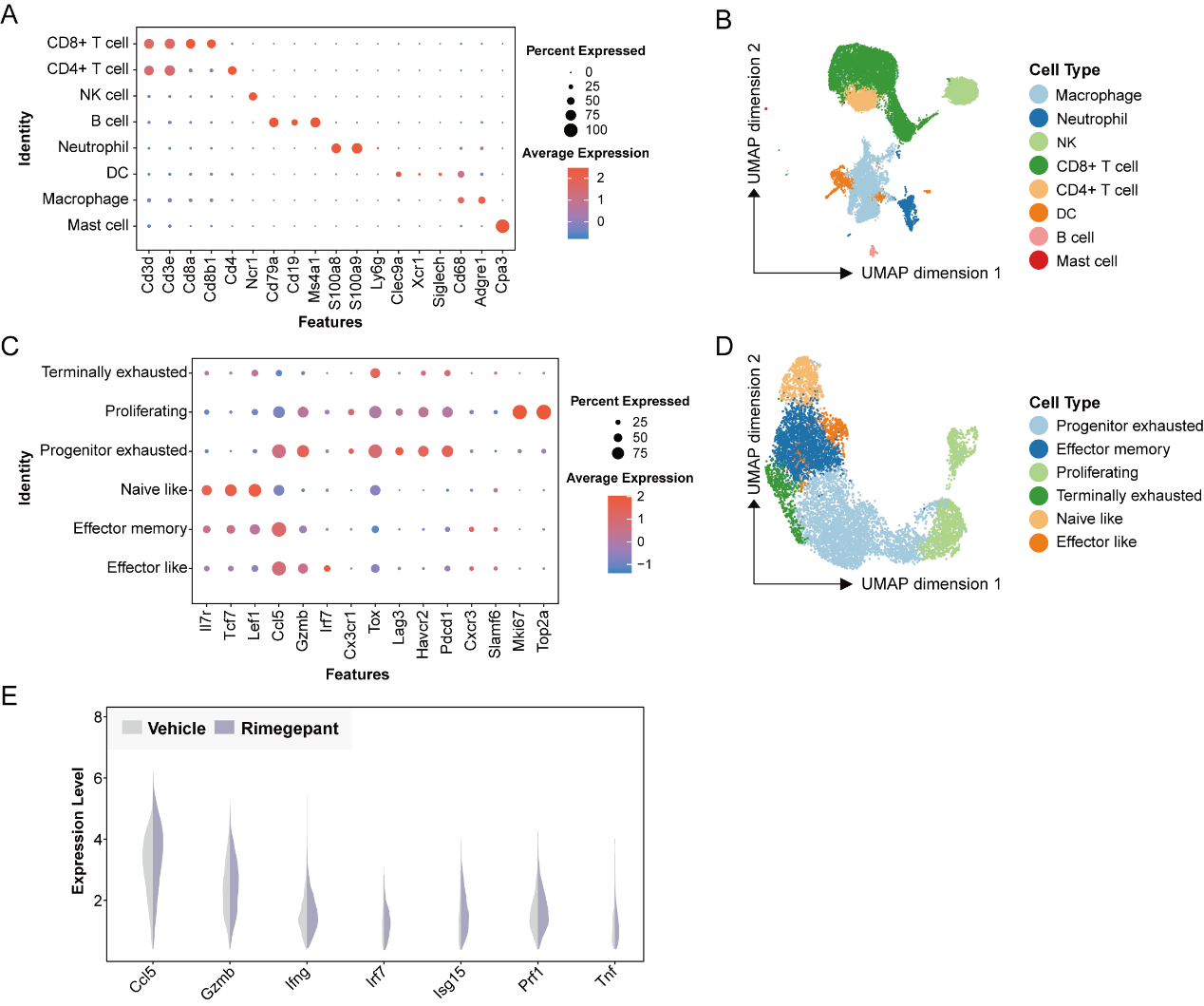


**Fig S6 | Single-cell RNA sequencing reveals immune remodeling induced by Rimegepant.**

(A, B) Comprehensive immune atlas of tumor-infiltrating immune cells. (A) Dot plot illustrating the expression of canonical lineage marker genes across identified immune cell populations. The dot size represents the percentage of cells expressing the gene, and the color intensity indicates the average expression level (scaled). (B) UMAP visualization of 25,018 tumor-infiltrating immune cells from vehicle- and Rimegepant-treated tumor, color-coded by cell type.

(C, D) Single-cell landscape and heterogeneity of CD8⁺ T cells. (C) Dot plot showing the expression of signature marker genes used to define functional subsets within the CD8⁺ T cell compartment. (D) UMAP visualization of 9,118 CD8⁺ T cells, annotated by their specific subset identities (e.g., Naive-like, Effector-like, Exhausted).

(E) Rimegepant promotes an effector-associated transcriptional profile. Violin plots comparing the expression levels of key effector and interferon-stimulated genes (Ccl5, Gzmb, Ifng, Irf7, Isg15, Prf1, and Tnf) between vehicle-treated (gray) and Rimegepant-treated (purple) groups.

Statistical Analysis: Single-cell RNA sequencing data were pre-processed using the Seurat (v4.0) pipeline, including quality control (filtering cells with low counts or high mitochondrial content), normalization (LogNormalize), and scaling. Data in (E) are presented as log-normalized expression values. Differential expression between groups was assessed using the Wilcoxon Rank Sum test. Sample size: n = 25,018 total immune cells and n = 9,118 CD8⁺ T cells derived from two biological conditions. Analysis was performed using R software.


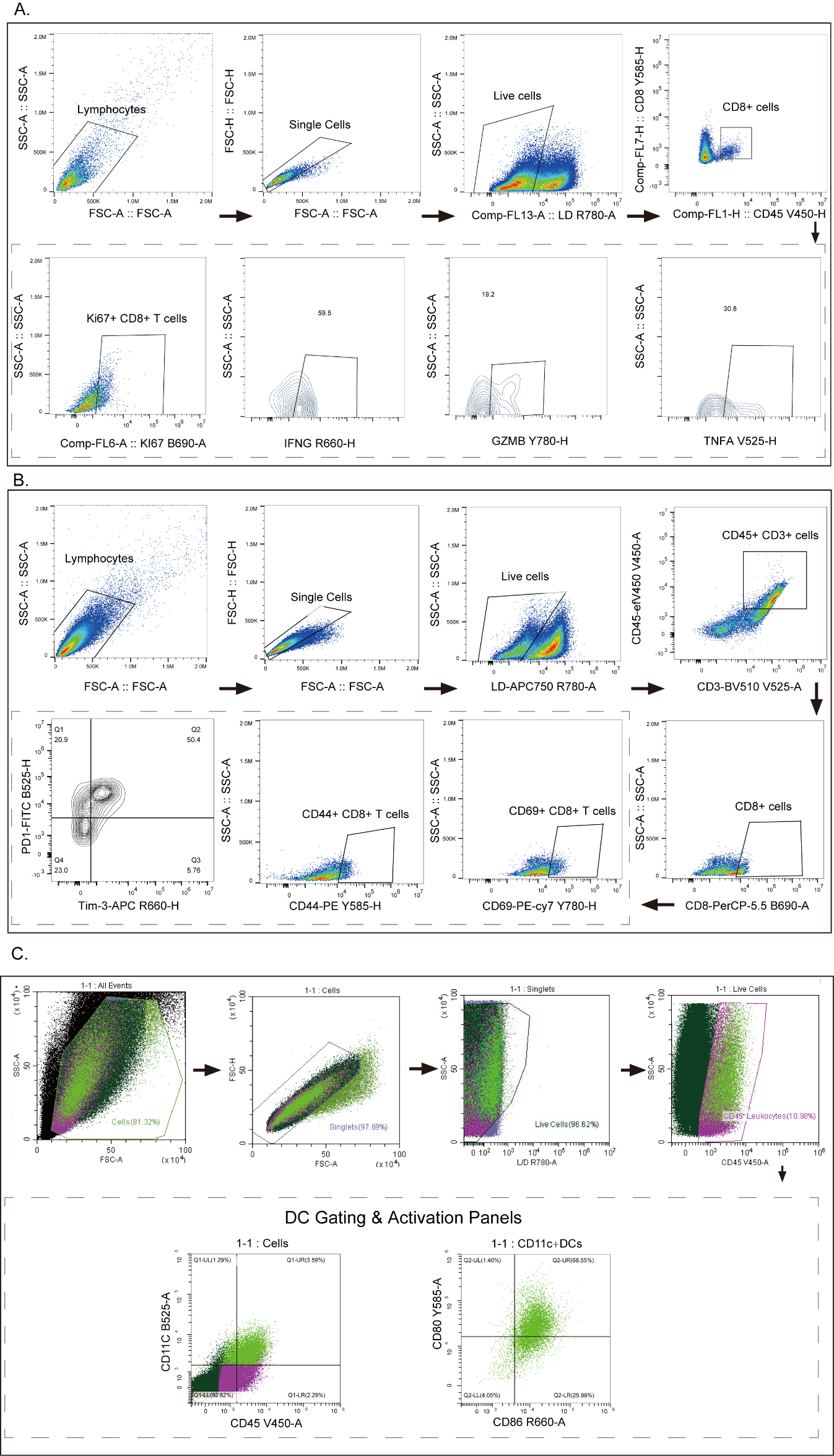


**Figure S7. Gating strategies for flow cytometry analysis of tumor-infiltrating lymphocytes and myeloid cells.** Representative flow cytometry gating hierarchies used to identify and characterize major immune cell populations within the murine tumor microenvironment, consistent with the gating strategy detailed in the Materials and Methods section. Positive gating boundaries were established using fluorescence minus one (FMO) controls.

(A) Gating Strategy for CD8⁺ T-cell Effector Function and Proliferation: Sequential gates were applied to isolate lymphocytes (FSC-A vs. SSC-A), single cells (FSC-A vs. FSC-H), and viable cells (LD R780-negative). Within the live CD45⁺ leukocyte population, CD8⁺ cells were directly gated to account for activation-induced CD3 downregulation following PMA/ionomycin stimulation. Subpopulations of Ki-67⁺, IFN-γ⁺, GZMB⁺, and TNF-α⁺ cells were subsequently quantified within this gate.

(B) Gating Strategy for CD8⁺ T-cell Activation and Exhaustion: Following the identical upstream sequential gating to isolate viable single cells (as described in panel A), CD45⁺CD3⁺ T cells were gated, followed by the isolation of CD8⁺ T cells. Sub-gating was then performed to quantify the expression of activation markers (CD44⁺ and CD69⁺) and the co-expression of inhibitory receptors (PD-1 and TIM-3) within the CD8⁺ T-cell gate.

(C) Gating Strategy and Activation Status of Dendritic Cells (DCs): Sequential gates were applied to isolate intact cells (FSC-A vs. SSC-A), single cells (FSC-A vs. FSC-H), and viable cells (LD R780-negative). Within the live CD45⁺ population, DC-enriched populations were identified as CD11c⁺ cells. The maturation and activation status of these cells were further quantified by the surface co-expression of CD80 and CD86.
