# Supplementary figures and images for: Pharmacologic Modulation of ARID3A with Rimegepant Reactivates Type I Interferon Signaling and Sensitizes Triple‐Negative Breast Cancer to PD‐1 Blockade
Source: Adv Sci (Weinh). 2026 Jun 2;13(40):e21541. doi: 10.1002/advs.202521541 (PMC13335518; doi:10.1002/advs.202521541)

FIG1B

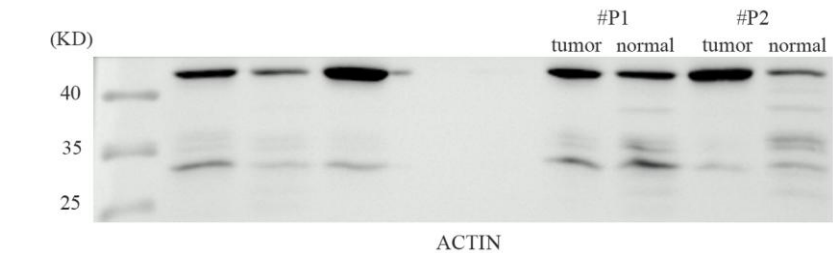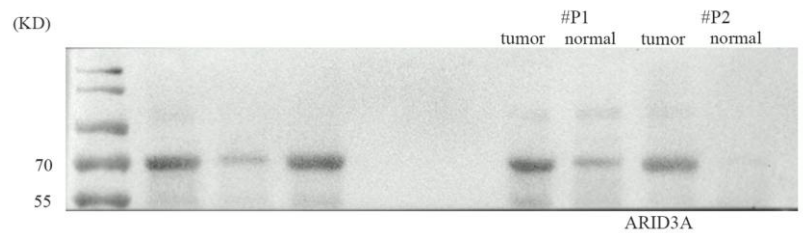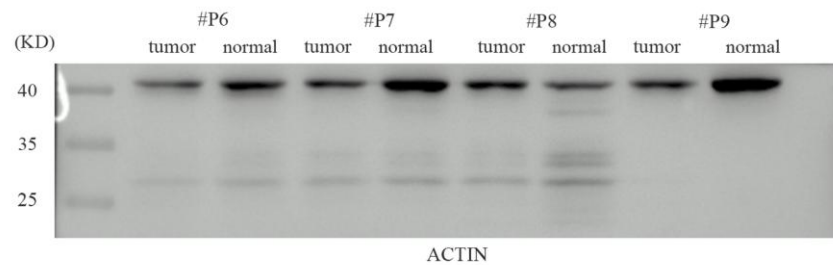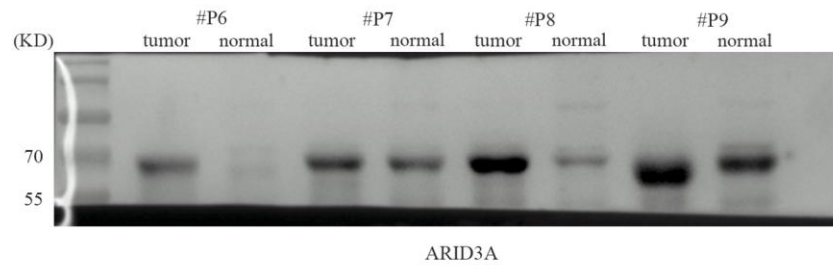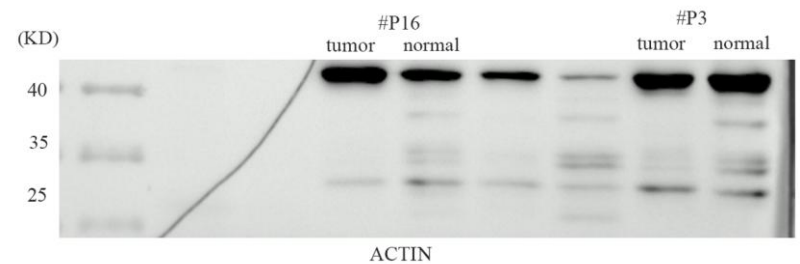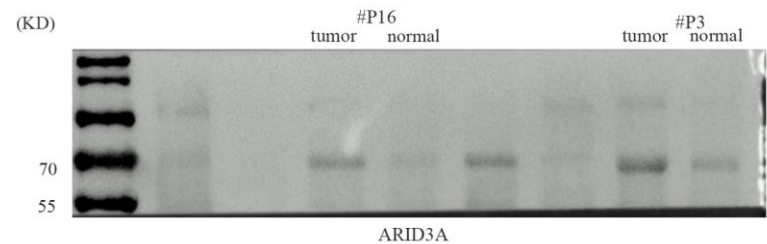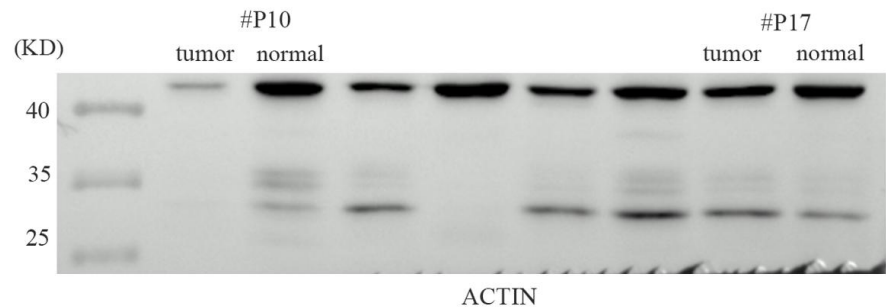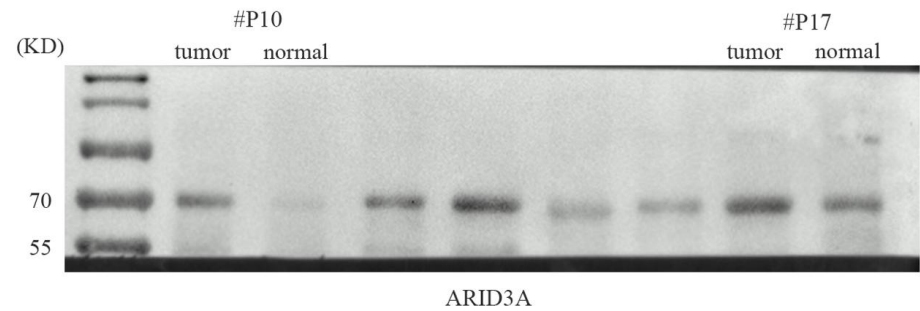

FIG1B

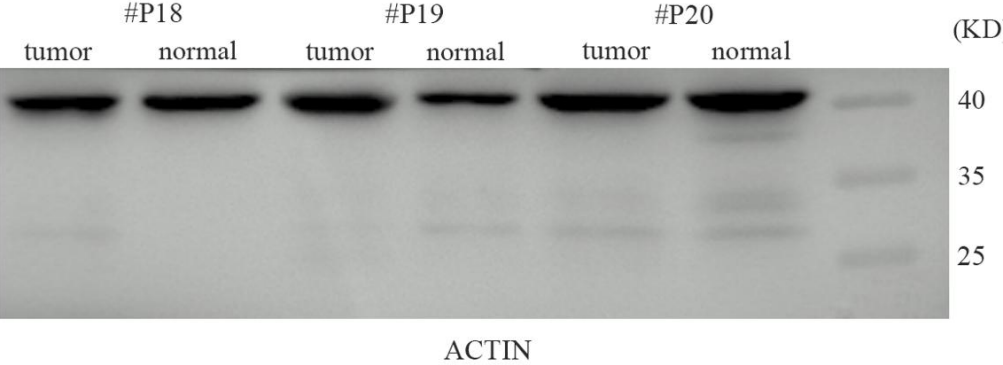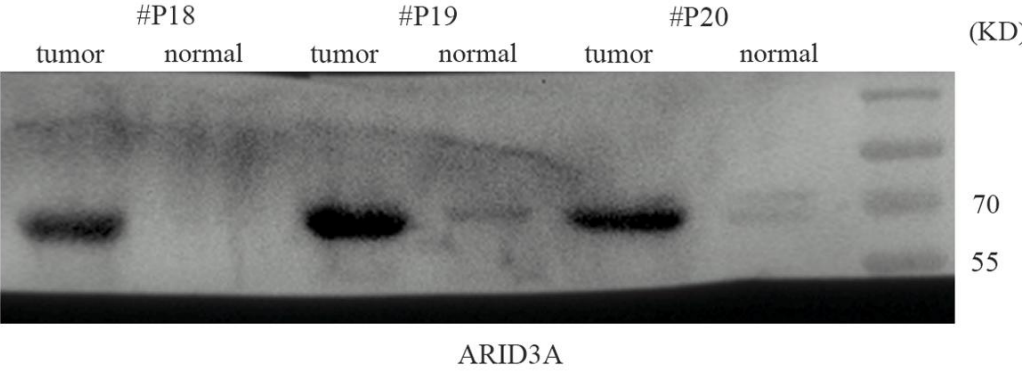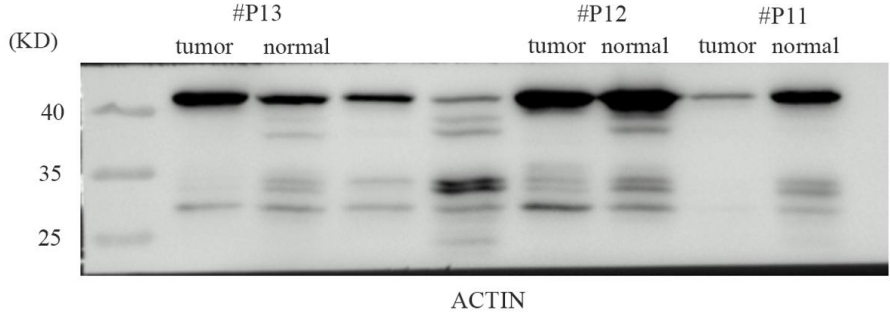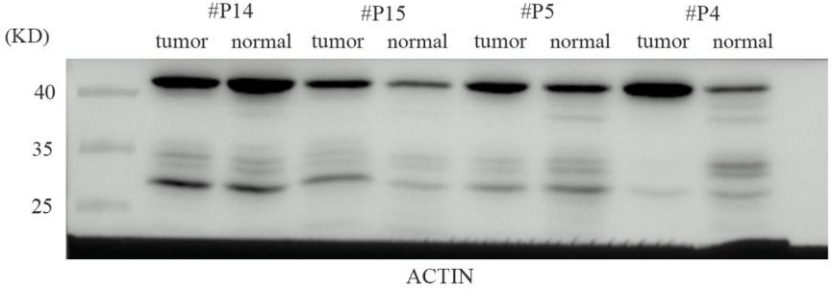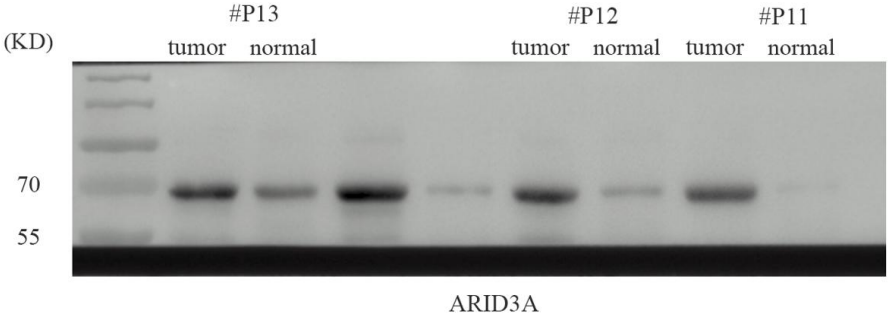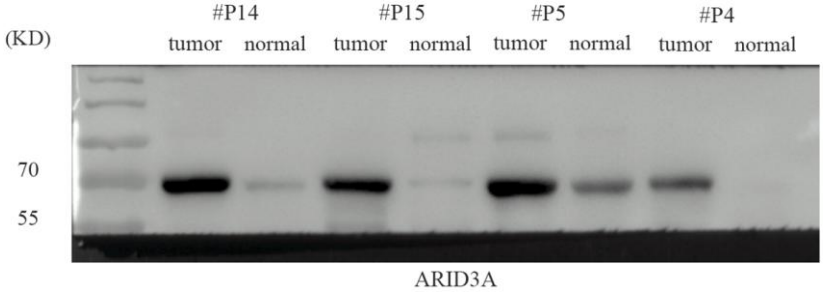

FIGS4E right 4T1

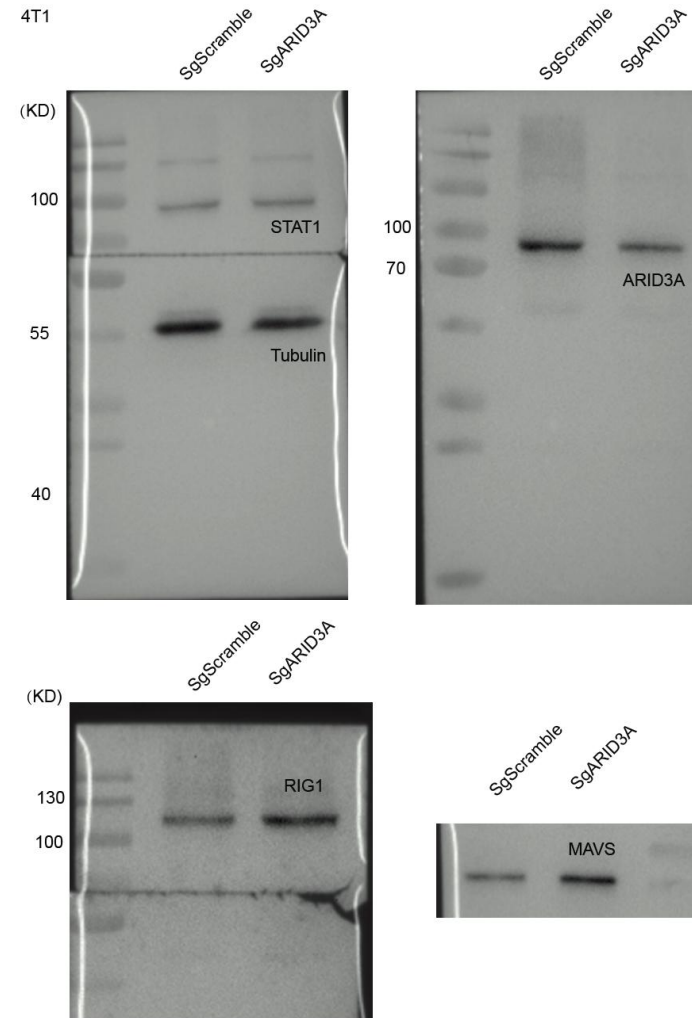

FIG4G left & FIG5M & FIGS4E left

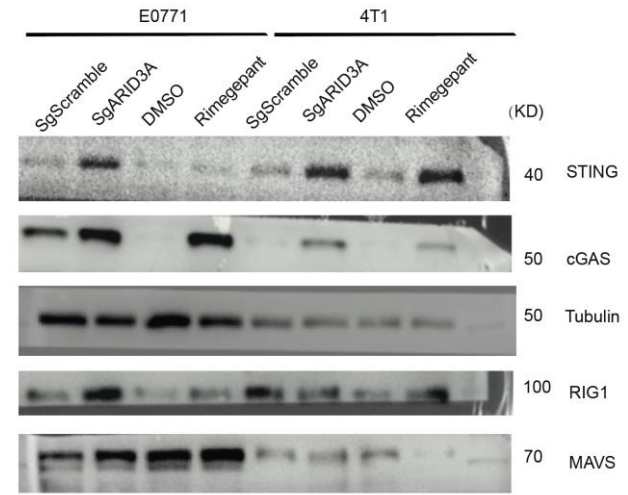

FIG4G right

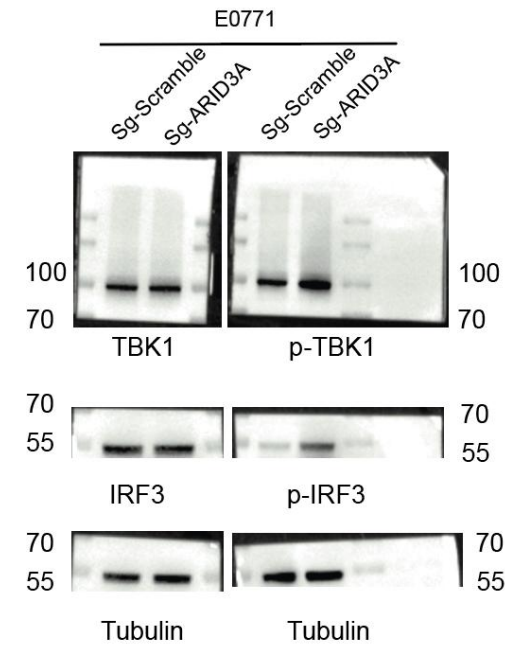

FIGS2B

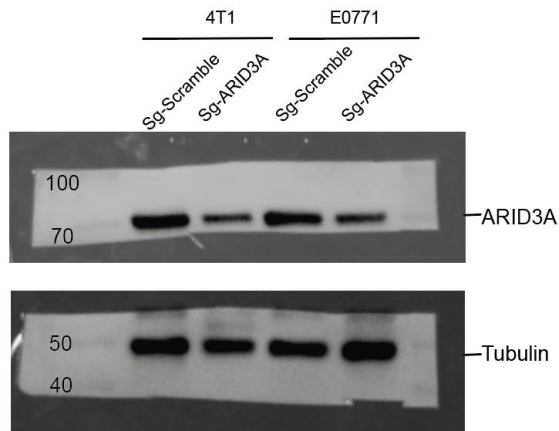

FIG5B

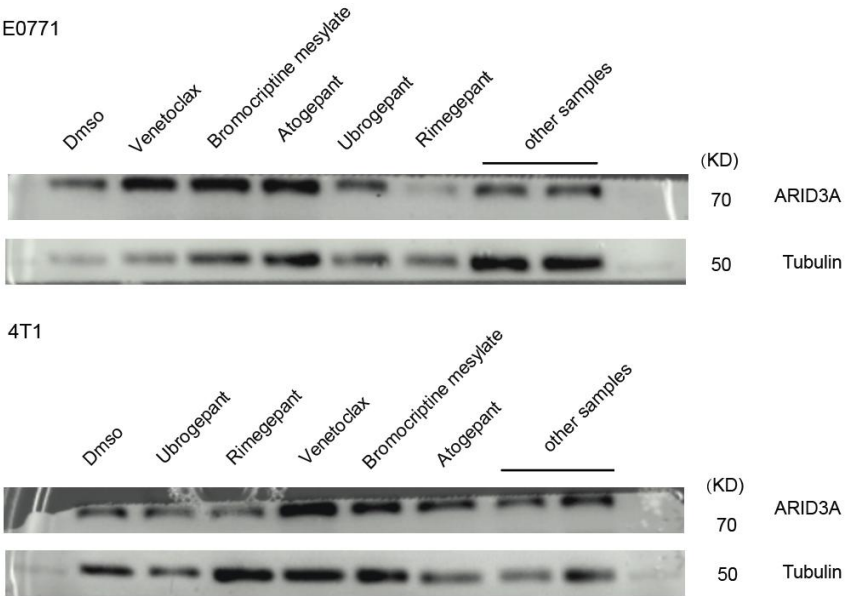

FIG5B

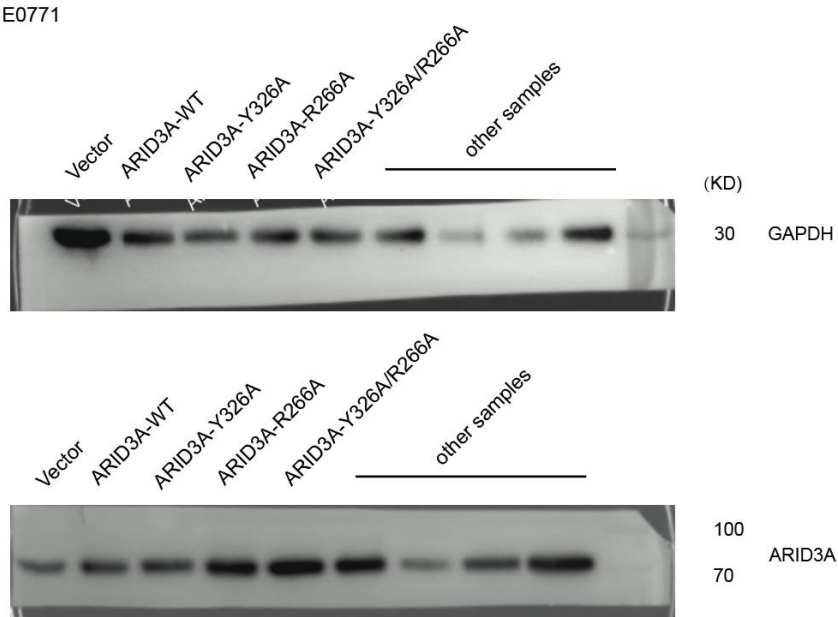

Supplement: Supplementary file 2 — Supporting File 2: advs75399‐sup‐0002‐DataFile.pdf. [file ADVS-13-e21541-s002.pdf]
